# Modeling the contribution of theta-gamma coupling to sequential memory, imagination, and dreaming

**DOI:** 10.3389/fncir.2024.1326609

**Published:** 2024-06-14

**Authors:** Gabriele Pirazzini, Mauro Ursino

**Affiliations:** Department of Electrical, Electronic, and Information Engineering “Guglielmo Marconi”, University of Bologna, Cesena, Italy

**Keywords:** theta-gamma coupling, episodic memory, hippocampus, encoding, retrieval, imagination, dreaming

## Abstract

Gamma oscillations nested in a theta rhythm are observed in the hippocampus, where are assumed to play a role in sequential episodic memory, i.e., memorization and retrieval of events that unfold in time. In this work, we present an original neurocomputational model based on neural masses, which simulates the encoding of sequences of events in the hippocampus and subsequent retrieval by exploiting the theta-gamma code. The model is based on a three-layer structure in which individual Units oscillate with a gamma rhythm and code for individual features of an episode. The first layer (working memory in the prefrontal cortex) maintains a cue in memory until a new signal is presented. The second layer (CA3 cells) implements an auto-associative memory, exploiting excitatory and inhibitory plastic synapses to recover an entire episode from a single feature. Units in this layer are disinhibited by a theta rhythm from an external source (septum or Papez circuit). The third layer (CA1 cells) implements a hetero-associative net with the previous layer, able to recover a sequence of episodes from the first one. During an encoding phase, simulating high-acetylcholine levels, the network is trained with Hebbian (synchronizing) and anti-Hebbian (desynchronizing) rules. During retrieval (low-acetylcholine), the network can correctly recover sequences from an initial cue using gamma oscillations nested inside the theta rhythm. Moreover, in high noise, the network isolated from the environment simulates a mind-wandering condition, randomly replicating previous sequences. Interestingly, in a state simulating sleep, with increased noise and reduced synapses, the network can “dream” by creatively combining sequences, exploiting features shared by different episodes. Finally, an irrational behavior (erroneous superimposition of features in various episodes, like “delusion”) occurs after pathological-like reduction in fast inhibitory synapses. The model can represent a straightforward and innovative tool to help mechanistically understand the theta-gamma code in different mental states.

## 1 Introduction

Episodic memory (EM) refers to remembering specific autobiographical events and their temporal context, a crucial cognitive function that enables us to navigate our daily lives. It is known that the hippocampus plays a crucial role in encoding and retrieving temporal episodes (Eichenbaum, 2017a). Working memory (WM) indicates the capacity to maintain and manipulate data for a short period, which is essential for many cognitive processes. While a traditional viewpoint assumes that this kind of memory just retains transitory data from the external world to drive imminent behavior, a broader perspective, which is becoming increasingly important recently (Hoskin et al., 2019; Beukers et al., 2021), considers a stricter relationship between working memory and other kinds of memory, especially the episodic one. Remarkably, Beukers et al. (2021) recently proposed that WM and EM collaborate to support complex behavioral patterns and that the relationship between EM and WM can parsimoniously explain much experimental evidence on working memory. However, how these functions are implemented in the brain remains a matter of active research. Consequently, substantial effort has been devoted to identifying the neural mechanisms behind them.

Recent work in neuroscience has indicated that neural oscillations with different frequencies and their phase relationships play a relevant role in many cognitive functions, including memory. In particular, fluctuations in the theta and gamma bands and their entrainment (the so-called theta-gamma code) are involved in WM and EM, as evidenced by the massive bibliography in the field (see Nyhus and Curran, 2010; Wang, 2010; Lisman and Jensen, 2013; Fries, 2015; Abubaker et al., 2021; Mysin and Shubina, 2022 for recent review papers).

An influential model hypothesizes that the theta-gamma entrainment is used for temporally coding objects or episodes within a sequence. In this model (Lisman and Idiart, 1995; Lisman, 2005; Lisman et al., 2005), cell assemblies that represent individual items fire in a highly synchronized way in the gamma band (> 30 Hz), while their phase defines the sequential order of these items within a slower theta rhythm (~ 4 Hz). Many results in rodents’ hippocampus reveal that high-frequency gamma oscillations are nested within slower theta oscillations (Soltesz and Deschênes, 1993; Belluscio et al., 2012; Colgin, 2016), fire at specific phases of the theta cycle, and, if an external input varies, exhibit a progressive phase shift (named precession phenomenon; Skaggs et al., 1996; Tsodyks et al., 1996; O’Keefe and Burgess, 2005). More recently, similar results have been confirmed in humans in the hippocampus and other parts of the cortex (Canolty et al., 2006; Chaieb et al., 2015; Heusser et al., 2016; Abubaker et al., 2021), suggesting that the theta-gamma code can have a broader role in realizing relational networks useful in many cognitive problems. In particular, theta-gamma may control the relationship between WM and EM and implement their reciprocal interactions.

Furthermore, brain rhythms can be altered in some neurological disorders, such as epilepsy, Alzheimer’s disease, or schizophrenia (Kitchigina, 2018; Yakubov et al., 2022); a study of their functional role can thus provide new indications and cues into the etiology of these pathological states. In particular, schizophrenia is characterized by abnormalities in theta and gamma oscillations (Spencer, 2008; Senkowski and Gallinat, 2015); the latter can contribute to symptoms and cognitive alterations by impinging on episodic and working memory.

Finally, a significant and partially new field of study concerns the role of these rhythms during sleep and the associated memory consolidation, particularly dreaming (Brodt et al., 2023), and during the imagination of new events in a wandering mind (O’Callaghan et al., 2021).

The previous analysis underlines the importance of a deeper understanding of gamma and theta oscillations’ role in memory and the necessity for a more systematic comprehension of the mechanisms subserving rhythmic cooperation in the brain. Neurocomputational models inspired by biology are playing an increasing role in this domain.

Most previous models of theta-gamma are devoted to simulating individual neuron behavior and studying place cells in the hippocampus (see Ursino et al., 2023, for a summary of these models). However, a different approach can be equally valuable, simulating neural activity on a larger mesoscopic scale and in a larger assembly of neural populations. This approach can better reveal some more general principles of functioning in which a distributed code involves large groups of neurons.

Mainly, neural mass models (NMMs) are a mathematical description of neural dynamics at a population level. These can simulate phenomena at different mesoscopic scales, ranging from local field potentials to an entire cortical region, using just a few state variables. Moreover, despite their simplicity (compared with detailed neuron models), they can include connectivity and synaptic dynamics, incorporate different families of neuronal populations, and account for the main non-linear phenomena in a straightforward but biologically founded way. Hence, they represent a good compromise between simplicity and biological reliability. In particular, NMMs helped to mimic the generation and transmission of brain rhythms in different frequency bands in physiological and pathological conditions (Wendling et al., 2002; David and Friston, 2003; Sotero et al., 2007; Bhattacharya et al., 2011; Cona et al., 2011; Cona and Ursino, 2015; Bensaid et al., 2019).

In recent years, we investigated the role of theta and gamma oscillations in memory with multi-layer neural mass models, analyzing the possibility of storing and recovering a sequence of episodes (Cona and Ursino, 2013) or representing trajectories in an allocentric space (Cona and Ursino, 2015). In our last work, we analyzed the role of theta and gamma rhythms in working memory (Ursino et al., 2023). In this model, a theta rhythm is generated internally to a first layer of NMMs due to reciprocal Hebbian auto-associative *excitatory synapses* that store the representation of objects, while the gamma rhythm is generated in a subsequent layer by creating Hebbian and anti-Hebbian *inhibitory syna*pses (i.e., synapses targeting fast GABA-ergic interneurons). Finally, the reconstruction of a sequence of items with an assigned temporal order is due to Hebbian hetero-associative *excitatory synapses* from a downstream to an upstream layer. After training, the model can maintain up to nine items simultaneously in memory by desynchronizing their gamma activity or, in a different functioning mode, replicate a sequence of items using a gamma rhythm nested inside a theta rhythm.

The central assumption of the previous model, however, was that both theta and gamma rhythms are generated *at a network level* as a consequence of Hebbian training.

Although this is a plausible hypothesis, several recent data support a different scenario, i.e., that theta oscillation in the hippocampus emerges from a complex interplay between local mechanisms and an external rhythmic drive (Etter et al., 2023; Mysin and Shubina, 2023; Robinson et al., 2023). In this regard, particular emphasis has been given to a role by the medial septum (MS). Manipulation of MS GABAergic neurons is critical in pacing theta rhythm (Robinson et al., 2023), whereas lesion of the MS or its pharmacological inactivation abolishes theta rhythmicity in the hippocampus (Boyce et al., 2016). Furthermore, in several conditions of cognitive relevance, such as quiet wakefulness or slow wave sleep, the hippocampus exhibits a non-theta activity. The importance of non-theta states for information processing in the hippocampus has been stressed by Mysin and Shubina in a recent review paper (Mysin and Shubina, 2023); suggesting that transitions between different hippocampal states occur within seconds. As further summarized in the “*Discussion*,” the previous results can be explained only with difficulty using the previous model. Hence, we need a more flexible model to explain theta rhythmicity and simulate the passage from theta to non-theta states faster and more straightforwardly.

Accordingly, this work presents a model alternative to the one described in Ursino et al. (2023) in which rhythms are generated independently of training. Each unit in the model might intrinsically oscillate with a gamma rhythm due to its fixed internal connections (specifically, local connections between pyramidal and fast GABAergic interneurons), while the theta rhythm is received from an external source (simulating the MS). In this more straightforward scenario, Hebbian synapses only reconstruct items from partial cues but are unnecessary for rhythm generation.

With this model, the interaction between WM and EM and the function of the theta-gamma code are assessed by training the model with sequences of episodes and investigating its capacity to recover them entirely from an initial cue. In addition, conditions simulating isolation from the external world (imagination and dreaming states, including non-theta slow-wave sleep) are also considered, as well as a dysfunction (such as schizophrenia) involving impairment of fast inhibitory connections. Finally, a comparison between the present and the older model is performed (see “*Discussion*”), and some testable predictions are proposed to validate the main hypotheses and discriminate the present model from previous ones.

## 2 Methods

The basic element of the network is the neural mass model, named “computational Unit” in the following. By linking several such Units, sophisticated networks can be realized to mimic complex cognitive processes.

### 2.1 The neural mass model

#### 2.1.1 The single computational Unit

It consists of four neural populations (pyramidal neurons, excitatory interneurons, and GABA-ergic inhibitory interneurons with slow and fast synaptic dynamics), reciprocally interconnected via feedback connections. The basic idea is that neurons of the same population exhibit common behavior (i.e., they share similar inputs and exhibit synchronized activity). Consequently, their global activity can be described using only a limited number of state variables.

Due to the interaction between excitatory and inhibitory populations, a single Unit can simulate different brain rhythms. In this work, parameters to individual Units have been assigned to mimic two possible intrinsic rhythms: a gamma rhythm (~ 40 Hz) or a theta rhythm (~ 4 Hz).

#### 2.1.2 Connections among Units

Besides generating an individual oscillation, Units can transmit or receive information via long-range connections originating from pyramidal neurons, thus realizing complex networks. Specifically, in the present model, each post-synaptic Unit can receive three different kinds of connections coming from pyramidal neurons in the pre-synaptic Units:

- Glutamatergic synapses targeting the pyramidal neurons of the post-synaptic Unit (excitatory connection). In the following, these will be represented with the symbol $Wp^{YX}$ , where superscripts *X* and *Y* stand for the pre-synaptic and post-synaptic layers, respectively.
- Glutamatergic synapses targeting the fast inhibitory interneurons of the post-synaptic Unit (thus realizing a bi-synaptic inhibitory connection: pyramidal→fast inhibitory→pyramidal). In the following, these will be represented with the symbol $Wf^{YX}$ .
- A third kind of synapses, represented in the following with the symbol $Af^{YX}$ , reaches post-synaptic fast inhibitory interneurons from pre-synaptic pyramidal neurons, but with very fast dynamics. These synapses, too, realize a bi-synaptic inhibition of the target pyramidal population.

The three connections described above play different roles in the model; hence are all necessary. Specifically, connections of type (*i*) are used to recover features in an object starting from an initial cue or to recover a new episode from a previous episode in a sequence. Briefly, they are essential to recuperating information in memory. Connections of type (*ii*) help synchronize features within the same episode so that all features in the same episode oscillate in phase with a gamma rhythm. Connections of type (*iii*) are essential to desynchronize features (therefore also named “desynchronizing synapses” in the following) in different episodes so that two episodes do not appear superimposed.

**Figure 1:**
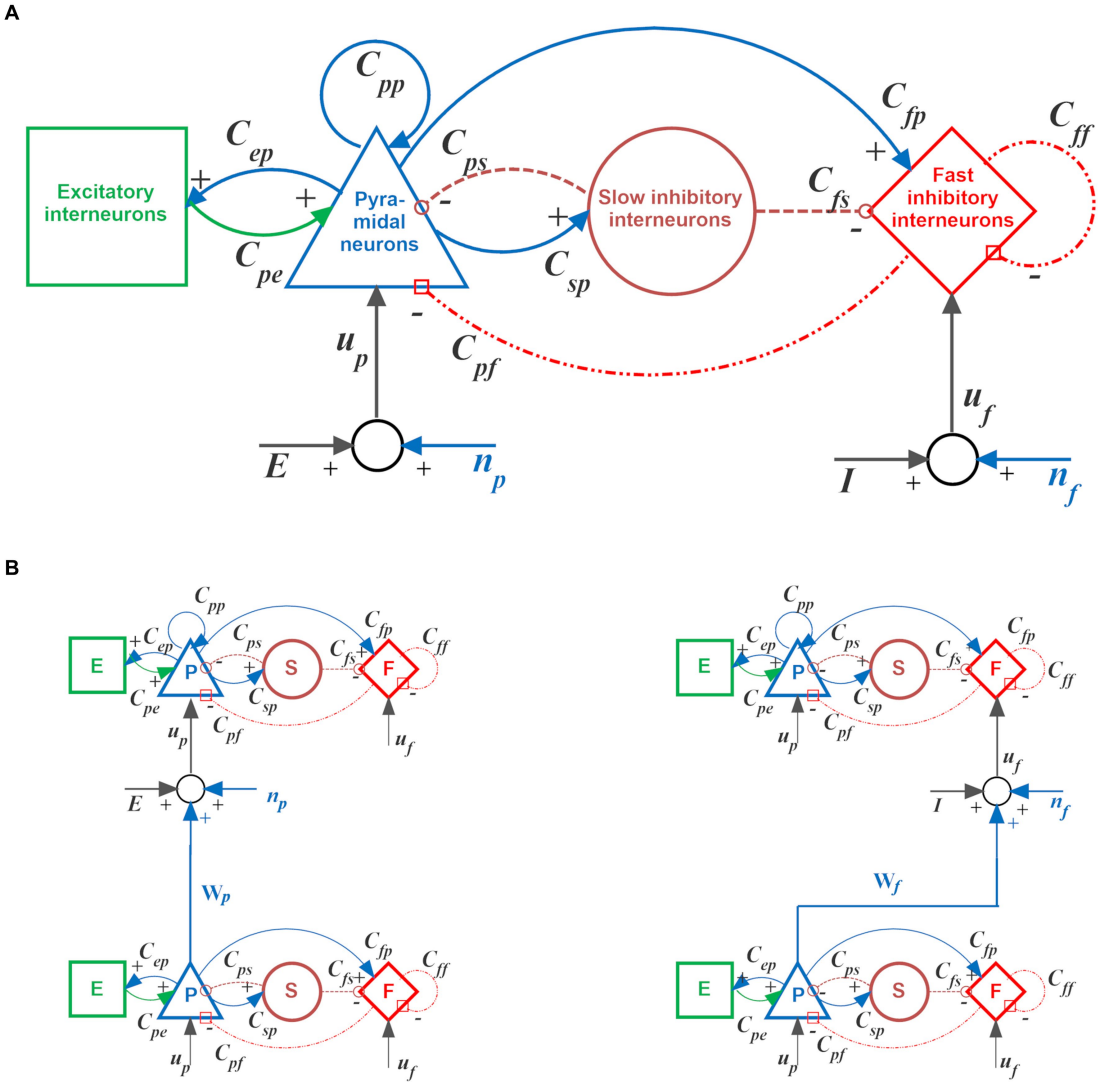
Single computational Unit and its possible interconnections. **(A)** Scheme of the neural mass model used to simulate the dynamics of each Unit. Blue and green continuous lines indicate glutamatergic excitatory synapses; red dash-dotted lines indicate GABAergic faster inhibitory synapses, while brown dotted lines indicate GABAergic slower inhibitory synapses. Symbols “*Cij*” denote the synaptic contacts among the neural populations, where the first and the second subscript designate the post-synaptic and pre-synaptic populations, respectively. Symbols “*u_p_*” and “*u_f_*” represent inputs to the pyramidal neurons and fast inhibitory interneurons, respectively. These inputs can came from synapses of pyramidal neurons from other Units or from the environment (“E” and “I”), or from noise (“*n_p_*” and “*n_f_*”). **(B)** An example of glutamatergic excitatory connection between Units, from the pyramidal neurons of the source Unit to the pyramidal neurons of the target Unit (named *W_p_* in the present model, *left column*), and an example of bi-synaptic inhibitory connection, from the pyramidal neurons of the source Unit to the fast inhibitory interneurons of the target Unit (which, in turn, inhibits pyramidal neurons in the target Unit, *right column*). In the present model, the latter may be either of glutamatergic *W_f_* type (as shown) or *A_f_* type (with almost instantaneous dynamics). For more details, see Supplementary material.

A detailed mathematical description of the single neural mass model and its interconnection mechanisms can be found in Supplementary material or in previous work by the authors (Ursino et al., 2010). The basic architecture of the computational Unit and connections between different Units are shown in Figure 1.

### 2.2 Structure of the model

**Figure 2:**
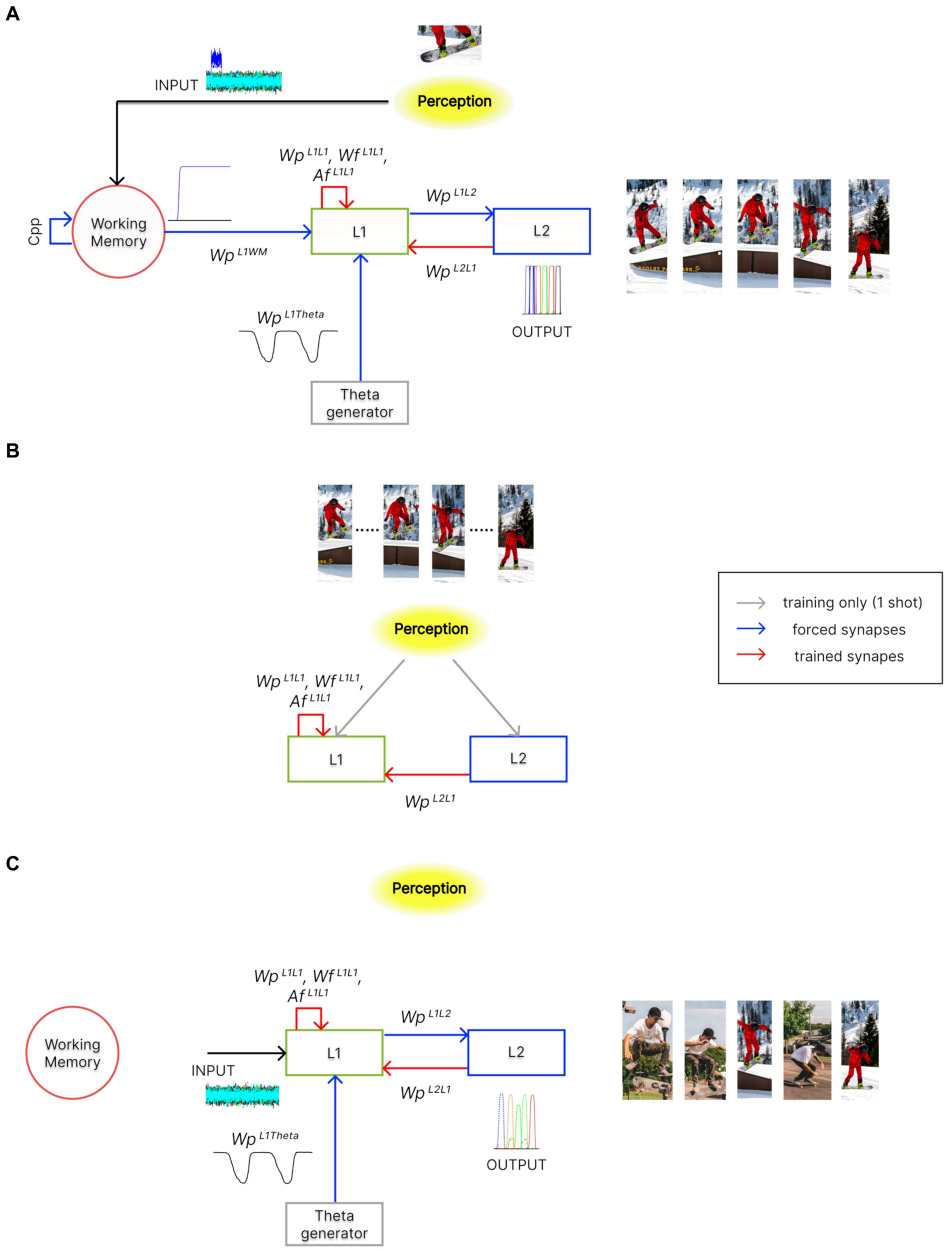
Architecture of the network in the different functioning modes. In the panels, red arrows indicate trained synapses, blue arrows fixed synapses, grey arrows the inputs used during training, and black arrows the inputs during the different functioning modes. Please note that pictures in these panels are used for representational purposes only. Indeed, features within an episode can also represent different sensory modalities or other more complex cognitive processes. **(A)** During the *Retrieval* functioning mode, the external perception (i.e., the result of integrating multisensory information from the outside world) indirectly reaches layer “L1” through the “WM” layer. Moreover, the activity in the “L1” layer is modulated by the “Theta generator,” which oscillates autonomously with the theta rhythm. “L1” can recall stored episodes, starting from their partial representation, through excitatory and inhibitory synapses, $Wp^{L1L1}$ and $Wf^{L1L1}$ . Additionally, excitatory synapses $Wp^{L1L2}$ , along with the desynchronizing synapses $Af^{L1L1}$ , allow the reconstruction of temporal sequences of episodes, which may also include common features. This overall mechanism occurs exploiting the phenomenon of theta-gamma coupling. The activity of the “L2” layer is considered the model’s output. **(B)** During the *Training* phase, the perception is now provided directly to the “L1” and “L2” layers, as in conditions of high-cholinergic drive. Two successive episodes of a time sequence are provided, once and simultaneously, to the two layers. This allows the formation of an auto-associative network within layer “L1” and a hetero-associative network from layer “L2” to “L1.” **(C)** During the *Isolation from the external world* operation mode, layer “L1” is disconnected from the “WM” layer and receives only high random input noise. Moreover, the strength of some synaptic connections can be altered (to simulate the effect of neurotransmitter changes or pathological conditions). The network can autonomously recall sequences of previously stored episodes (“Imagination”), create new oneiric combinations of them (“Dreaming”), or exhibit superimpositions of episodes as in neurocognitive disorders (“Schizophrenia”).

The model consists of three layers of multiple computational Units (in the following named “WM,” “L1,” and “L2”), oscillating with a gamma rhythm. In addition, a single external Unit (called “Theta generator”) generates a theta rhythm. A block diagram of the three-layer arrangement, along with its operation modes (see also “*Functioning modes*” below), is shown in Figure 2.

Each Unit in each layer codes for a single *feature*, and these features are replicated identically in each layer. Hence, the layers have the same number of Units. In the authors’ minds, these features may represent any sensory experience (i.e., visual, olfactory, auditory, etc.) or even a more complex cognitive aspect (e.g., an emotion, a motor gesture, etc.). An *episode* consists of a variable number of features occurring together, while a temporally ordered succession of episodes represents a *sequence*.

A feature is considered correctly recovered when the spike density of pyramidal neurons in the corresponding Unit oscillates with a gamma rhythm. Furthermore, each episode is considered correctly recovered when all the Units representing its component features oscillate in a highly synchronized manner.

In particular, each layer of the network receives excitatory forward synapses of type (*i*) (see above) from the previous layer (linking each Unit that codes for a specific feature with the corresponding Unit coding the same feature in the next layer). Furthermore, all Units in layer “L1” receive an excitatory synapse from the “Theta generator.” These synapses are fixed and thus not subject to training.

All other synapses implementing feedback associative mechanisms among Units are plastic and trained through Hebbian and anti-Hebbian learning mechanisms (see “*Training of the network*” below).

In the following, the layers that make up the model architecture will be described in greater detail, and some assumptions on their possible anatomical location will also be given.

#### 2.2.1 The working memory layer

The “WM” layer is the first module of the network, consisting of 75 Units (one for each distinct feature used in this work). In the normal *Retrieval* functioning mode, Units coding for some particular features are excited with a high input (in addition to noise), signaling that the corresponding feature has been detected in response to an external stimulus. The other Units receive only noise (with zero mean value) and are silent, signaling that this feature has not been detected. No lateral connection exists between features in this layer. In fact, the aim of “WM” is just to maintain information in memory even when the external input ceases by protecting it from interference and updating it as soon as a new input arrives. Our model realizes this behavior, which is typical of working memories (Balaban and Luria, 2017), through an auto-excitatory loop for pyramidal neurons (described with the internal parameter “*Cpp*,” see Supplementary material). Furthermore, this auto-excitation is reset to zero once a new input reaches the “WM” layer (see also Ursino et al., 2023).

A natural location for the “WM” layer is the prefrontal cortex (PFC). In fact, numerous works in the literature suggest a neural interaction between the ventromedial PFC and the hippocampus during memory tasks (Eichenbaum, 2017b; Campbell et al., 2018; Barry et al., 2019). An auto-excitatory loop to maintain information in working memory can be realized via a basal ganglia-thalamus gating mechanism that allows for selective updating of the prefrontal cortex, as discussed by Nir-Cohen et al. (2020). In particular, these authors discuss the involvement of these structures in opening the gate and replacing the present content in WM with new information.

The activity of “WM” is then transmitted to the second layer of the network, the “L1” layer.

#### 2.2.2 Layer 1

Layer “L1,” as the previous, consists of 75 Units, initially disconnected from one another. We assume that, in the normal *Retrieval* operating mode, all Units of the “L1” layer are inhibited but receive rhythmic disinhibition from the “Theta generator,” realizing a theta-gamma code.

After *Training*, this layer behaves as an auto-associative network, capable of retrieving the complete information of an episode starting from a partial representation. To this end, excitatory ( $Wp^{L1L1}$ ), inhibitory ( $Wf^{L1L1}$ ), and desynchronizing ( $Af^{L1L1}$ ) synapses linking the different Units within “L1” are trained, during a learning phase, using Hebbian and anti-Hebbian training mechanisms (see *“Training of the network”* below). After *Training*, the various features become interconnected with each other, depending on the particular events previously experienced, and the balance between excitation and inhibition allows all features of an episode to be recovered and to oscillate in synchronism with the gamma rhythm (~ 40 Hz).

The “L1” layer could be located in the CA3 region of the hippocampus, where the presence of auto-associative solid feedback synapses is well documented (Bliss and Collingridge, 1993) or it can simulate a larger portion, including the perirhinal/entorhinal regions (Eichenbaum, 2017b) as well. The presence of fast synapses can be justified by the presence of more rapid AMPA synapses or by plastic gap junctions. The latter have been recently documented between principal hippocampal cells (Schmitz et al., 2001; Molchanova et al., 2016).

#### 2.2.3 Theta generator layer

In the present model, contrarily to our previous work, the theta rhythm is not generated internally to a layer but is produced externally by another Unit, named “Theta generator,” and then transmitted to each Unit in “L1” through a glutamatergic excitatory synapse, thus disinhibiting their activity. This simple portion of the model was implemented through a single neural mass model, autonomously oscillating in theta rhythm (~ 4 Hz) by providing particular values of the internal parameters of the populations (see Supplementary material).

Recent literature shows that the medial septum can play a role in the genesis of theta waves in the hippocampus (Pignatelli et al., 2012). Another hypothesis is that a theta-rhythmic signal may resonate throughout Papez circuit, which involves different structures, including the mammillary bodies, the mammillothalamic tract, the anterior thalamic nuclei, the cingulate cortex, and the parahippocampal gyrus (Vertes et al., 2001).

#### 2.2.4 Layer 2

Finally, the “L2” layer, together with “L1,” realizes a hetero-associative network to correctly reconstruct a complete sequence of episodes, starting from an initial one. During *Training* (see below), excitatory connections ( $Wp^{L1L2}$ ) are formed from Units in “L2” to Units in “L1,” specifically between the features of a particular episode in “L2” and the features of the subsequent episode in “L1.” This network also consists of 75 neural mass models.

A possible location for the “L2” layer may be in the CA1 cells of the hippocampus. In fact, CA1 activity seems essential for the temporal memorization of a chain of episodes (Hoge and Kesner, 2007; Mankin et al., 2012). The presence of bidirectional synapses between CA3 and CA1 can occur through the lateral entorhinal cortex, as shown in Eichenbaum (2017b). Moreover, the presence of Granger causality from CA1 to CA3 cells has been detected recently (Sandler et al., 2015).

### 2.3 Training of the network

All feedforward synapses in the model (i.e., from one layer to the subsequent one and from the “Theta generator” to the “L1” Units) are fixed. This simplifying assumption will be critically discussed in the last section. Conversely, feedback synapses are trained. As stated above, these are the internal synapses within the layer “L1” (excitatory connections $Wp^{L1L1}$ , inhibitory connections $Wf^{L1L1}$ and desynchronizing connections $Af^{L1L1}$ ) and the synapses from “L2” to “L1” layer (excitatory connections $Wp^{L1L2}$ ).

During *Training*, simulating an encoding phase, we provided the information of each episode to layers “L1” and “L2” by stimulating pyramidal neurons and fast inhibitory interneurons of the corresponding features for 250 ms. In particular, we used the following strategy to memorize a sequence of *N* episodes in an assigned temporal order. Naming the episodes in a sequence as: *Ep_1, Ep_2*,…, *Ep_N*, during each training step the following inputs are provided:


“L1”:Ep_k;“L2”:Ep_k−1withk=1,2,…N+1

In which *Ep_0* and *Ep_(N + 1)* represent “null” episodes, i.e., no feature is stimulated. The direct use of inputs to “L1” and “L2” can be justified based on experimental evidence showing that, during the encoding of memory content in the hippocampus, high levels of the neurotransmitter acetylcholine facilitate a direct connection between the cortex and the hippocampal nuclei, in particular CA1 and CA3, while connections between CA1 and CA3 are temporally suppressed (Hasselmo, 2006; Decker and Duncan, 2020). Notably, each pair of episodes is provided only once as network input, leading to successful storage.

Several studies suggest how the transmission of current sensory information from the entorhinal cortex to the hippocampus may be facilitated by a faster gamma rhythm (> 40 Hz) (e.g., Bieri et al., 2014 and Newman et al., 2013). However, some recent findings challenge this hypothesis (see, for example, Yamamoto et al., 2014), and further evidence is needed to better understand the functional significance of fast gamma in the entorhinal-hippocampal network (Colgin, 2015). Therefore, in the current model, we decided to perform a simple *Training* where incoming information constantly excites the activity of pyramidal and GABAfast neurons in the “L1” and “L2” layers. In the future, an improved model could take into account these aspects, e.g., by implementing a “fast” gamma rhythm driven by higher sensory/integrative areas to the entorhinal cortex during encoding.

All the glutamatergic synapses (i.e., the excitatory $Wp^{L1L1}$ , the inhibitory $Wf^{L1L1}$ , and the excitatory $Wp^{L1L2}$ ) are trained with a Hebb rule, based on the correlation between the pre-synaptic and post-synaptic activities. Conversely, the fast synapses $Af^{L1L1}$ , used to desynchronize different episodes, are trained with an anti-Hebbian mechanism. The latter allows us to obtain an extremely rapid desynchronization so that features belonging to different episodes inhibit themselves during the reconstruction of a sequence. A detailed mathematical description of the two different learning rules can be found in Supplementary material.

We also implemented a normalization mechanism, i.e., the sum of synapses of a specific type entering a computational Unit must not overcome a given threshold. This simulates the physiological limitation of neurotransmitters and allows the network to work with episodes consisting of a different number of features (4, 5, or 6 in the present paper, see Supplementary material) while maintaining a similar behavior.

**Figure 3:**
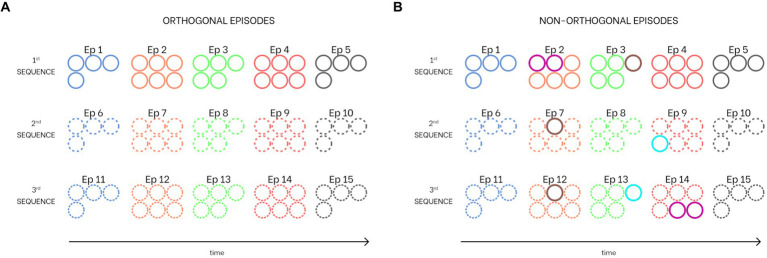
Combination of sequences learned from the network. Each circle corresponds to a particular feature (i.e., the number of circles for each episode corresponds to the number of features that constitute it). Common features between episodes are indicated with different colored circles. **(A)** Three sequences with orthogonal episodes (i.e., episodes without shared features). **(B)** Three sequences with shared features (i.e., non-orthogonal episodes). Specifically, the second episode of the first sequence has two features in common with the 14th episode (fourth of the third sequence), the third episode of the first sequence has one feature shared with both the seventh episode (second of the second sequence) and the 12th episode (second of the third sequence), and the ninth episode (fourth of the second sequence) has one feature in common with the 13th episode (third of the third sequence). All colors and hatching styles are consistent with those used in all figures in this work.

Since auto-associative networks exhibit optimal behavior in the presence of orthogonal patterns, and, moreover, many authors claim that the dentate gyrus orthogonalizes input patterns before they are stored in CA3 cells (Knierim and Neunuebel, 2016; Kesner, 2018), we performed a first training procedure using three sequences composed of five orthogonal episodes each (i.e., five episodes per each sequence without any common feature, see Figure 3A). However, our model exhibits a richer and more complex behavior (see *“Isolation from the external world”* below) in the presence of some shared features. For this reason, we also performed a second training procedure, still with five episodes, but now with one or two shared features per episode (i.e., from 20 to 35% of the total episode can be shared). Figure 3B illustrates this particular set of non-orthogonal sequences. Specifically, Episode 2 (belonging to the first sequence) shares two features with Episode 14 (third sequence), Episode 3 (first sequence) has one feature in common with both episodes 7 and 9 (second and third sequence), and Episode 9 (second sequence) has one feature in common with Episode 14 (third sequence). Since in the present simulations we are using either orthogonal episodes or episodes which share only a few features, some sort of previous orthogonalization is implicitly assumed. In the future, a new layer could be implemented to explicitly analyze the role of the dentate gyrus in orthogonalization of input patterns and the processing of episodes sharing common features.

Of course, longer sequences (with more than five episodes) can be trained equally well. However, the length of recalled sequences in this model would still be a maximum of five episodes due to the implemented mechanism of theta-gamma coupling. Similarly, more complex episodes (consisting of more than six features) can be correctly encoded and retrieved by the network. We tested that the model is still able to manage up to 50 features per episode correctly (unpublished results).

### 2.4 Functioning modes

The model has been used with three different operating modes: (i) “Training,” (ii) “Retrieval,” and (iii) “Isolation from the external world.”

- As described above, in the *Training* modality, the network learns all the associative synapses with Hebbian and anti-Hebbian rules (Figure 2B).
- In the *Retrieval* mode, the network, after *Training*, can complete an episode starting from a single input feature and reconstruct a temporal sequence of different episodes. Specifically, the input from the outside world is retained in memory in the “WM” layer and then transferred to the “L1” layer. If this input represents one of the features of a specific stored episode, the “L1” layer reconstructs the overall episode. Then, the integrated action of the “L1,” “L2” layers, and “Theta generator” recovers the overall sequence of episodes by exploiting the theta-gamma coupling (Figure 2A). The sequence is repeated at each theta cycle until a reset signal arrives to the “WM.”
- In the modality *Isolation from the external world*, the network is isolated from the environment by disconnecting the “L1” layer from the “WM” layer (see Figure 2C), and all neurons in “L1” receive a strong positive input noise (with uniform distribution ranging between a minimum and a maximum) in addition to the previous zero mean value noise given in the *Retrieval* mode (see Supplementary material). To simulate some special conditions of the human mind (imagination and dreaming), or pathological cases (like schizophrenia), the amplitude of noise and the strength of synapses has been changed compared with the *Retrieval* to account for a possible effect of neurotransmitters (for instance, during sleep) or pathological changes. Specifically:
  - in the condition named “*Imagination,”* the noise level to “L1” neurons has been increased, still maintaining the same synapse values as in the *Retrieval* phase;
  - in the condition named *“Dreaming,”* noise to “L1” neurons has been further increased, and all synapses have been reduced by 2/3. Furthermore, three different possibilities were considered: the first two with the presence of theta modulation (but different gamma frequencies), the third not; and
  - in the condition named “*Schizophrenia*,” noise is still increased and all fast synapses Af within the “L1” layer have been reduced to simulate some impairment in fast inhibitorymechanisms.

A deeper justification of all these changes will then be made in the “*Discussion”* section.

### 2.5 Quantitative model assessment

To quantitatively evaluate the model performance, we compared the activity of each pyramidal neuron in layer L2 to an assigned threshold. We consider that an episode is wholly recovered within a given theta cycle if all features of that episode are simultaneously above the threshold for at least one integration step. Furthermore, we say that a sequence is recognized within a theta cycle if its episodes are recognized (according to the previous criterion) in the correct order. In the following, the threshold will be given a value as high as 70% of the maximum activity of pyramidal neurons (i.e., 0.7*5 = 3.5).

Following the previous criterion, the model’s performance has been assessed by performing 10 simulations for each sequence, with a duration of 1.0 s, and using a different seed for the noise (i.e., 30 simulations were performed). During each simulation, a feature of the first episode was given as an input, and the capacity to recover the overall sequence was evaluated during each theta cycle. The results are expressed as the percentage of correct episode retrieval, assessed separately for each episode in the sequence, from the first to the last.

The model assessment was first performed with the basal parameter values, using both the orthogonal and the non-orthogonal sequences, to evaluate the role of shared features. Since an essential characteristic of the present model is that theta and gamma oscillations are autonomously generated within the Units, subsequently, we evaluated model performance by changing the frequency of the theta rhythm generated by the “Theta generator” and then the frequency of the gamma rhythm generated in the “L1” and “L2” layers. This is a critical analysis since brain rhythm frequencies can be altered in different *in vivo* conditions. Finally, we also tested the robustness of the networks by changing the parameters involved in synaptic training mechanisms.

## 3 Results

### 3.1 Encoding and retrieval of temporal sequences

#### 3.1.1 Orthogonal sequences

**Figure 4:**
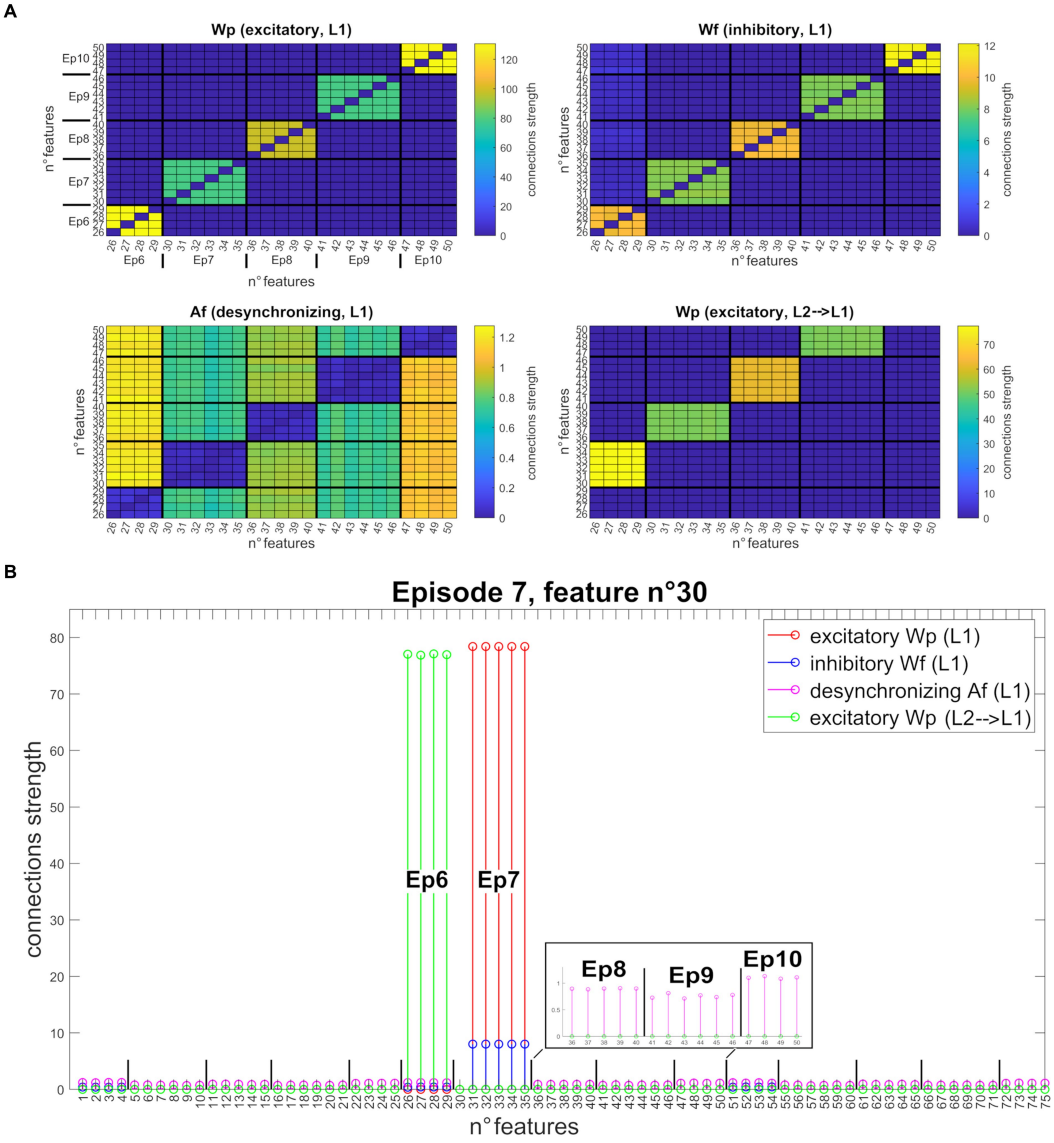
Synapses obtained after network training (orthogonal features). **(A)** Strength of the four different types of synapses (from top to bottom, left to right: $Wp^{L1L1}$ , $Wf^{L1L1}$ , $Af^{L1L1}$ , $Wp^{L1L2}$ ), linking all features belonging to the second sequence (five episodes, features from 25 to 50). Each row of the matrix represents a post-synaptic feature, and each column a pre-synaptic feature. For better understanding, the different episodes are marked in the upper left figure. The *right and left upper figures* show that each feature of a particular episode is linked to the other features of the same episode (via excitatory and inhibitory connections). On the *bottom left figure*, features of a particular episode inhibit all features belonging to different episodes, via desynchronizing connections. On the *bottom right figure*, features of a specific episode receive excitatory connections from the features of the temporally preceding episode. Interestingly, due to the normalization mechanism, the strength of connections between Units differs according to episode richness. **(B)** Set of connections entering the post-synaptic Unit n° 30 (that codes for the first feature of episode 7) from all other Units in the network. All synapses are shown on the same graph through different colors ( $Wp^{L1L1}$ in red, $Wf^{L1L1}$ in blue, $Af^{L1L1}$ in purple, and $Wp^{L1L2}$ in green). The episodes belonging to the second sequence are explicated in the graph. The 30th feature receives excitatory and inhibitory synapses from all other features in the same episode (from 31 to 35), excitatory synapses from all features of the preceding episode (the sixth, from 26 to 29), and desynchronizing synapses from all other stored episodes (even of different sequences). To better highlight these latter connections, the $Af^{L1L1}$ synapses received from the features of episodes 8, 9, and 10 are zoomed in the black box.

Figure 4 shows the synapses obtained by training the network with three different sequences of five episodes, all orthogonal. Specifically, Figure 4A shows the strengths of connections between all features of the second sequence (Units from 25 to 50). After *Training*, the “L1” layer realizes an auto-associative network due to the formation of excitatory $Wp^{L1L1}$ and inhibitory $Wf^{L1L1}$ synapses between all features of the same episode (top left and right figures). In addition, desynchronizing synapses $Af^{L1L1}$ are created in the same layer between features of different episodes (bottom left figure). Finally, the bottom right figure shows the excitatory $Wp^{L1L2}$ synapses from “L2” to “L1” layer that realize a hetero-associative network linking one episode in “L2” to the temporally following episode in “L1.” Interestingly, thanks to the normalization mechanism, the weight of synapses entering each Unit decreases with the richness (i.e., the number of features) of its episode. To better clarify the synapse pattern, Figure 4B shows the strength of connections entering Unit 30 (belonging to the seventh episode, second sequence), corresponding to row number 30 of each figure in Figure 4A. This feature receives excitatory and inhibitory synapses from all other features of Episode 7, desynchronizing synapses from all other stored episodes, and excitatory feedback synapses from Episode 6 (the temporally preceding one).

**Figure 5:**
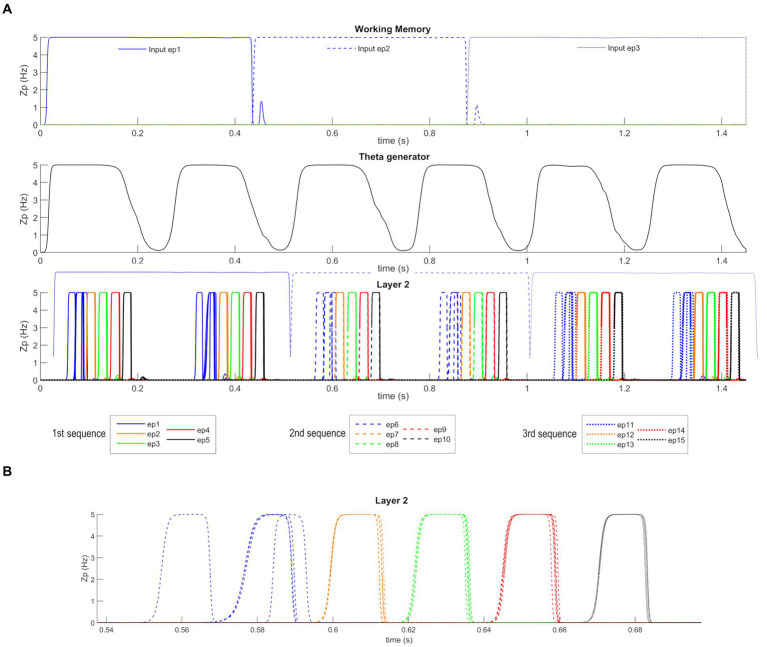
Retrieval operation mode (orthogonal features). **(A)** Temporal activity of the pyramidal neurons of three layers of the network: “WM,” “Theta generator,” and “L2,” during *Retrieval* operation mode, in the presence of orthogonal episodes. A value of 5 Hz is the maximum discharge value of the Units in the model. During the simulation three different features, belonging to the first episode of each sequence (Episode 1, Episode 6, and Episode 11), are provided for a short time as input to the network. The five episodes constituting each sequence are represented through a list of colors (in order blue-orange-green-red-black), while the different sequences are distinguished through different hatching styles (Sequence 1: continuous, Sequence 2: dashed, Sequence 3: dotted). The Working Memory layer can maintain the information of the input feature even when no longer provided and updates itself as soon as a new input is given. The “Theta generator” oscillates autonomously with a theta rhythm and acts as a rhythmic disinhibitor for the “L1” layer. The activity of the “L2” layer is considered the network output (“L1” activity is not shown for brevity since it behaves similarly but more irregularly). The network reconstructs the first episode of the sequences starting from a single feature and recalls the subsequent episodes by oscillating with a gamma rhythm modulated by the slower theta rhythm. In the third row, the output of the “WM” layer is reported again in transparency to better show the network update. **(B)** A zoom of the second sequence of episodes (from Episode 6 to Episode 10), between 0.54 and 0.7 s. The first feature of the sixth episode recalls all other features of the same episode, which in turn recalls the first feature again, slightly delayed. Then, each episode recalls all features of the following episodes, highly synchronized with a gamma rhythm. The sequence stops after half of the theta cycle.

After *Training*, the network can work in the *Retrieval* mode operation; that is, it can recall an entire ordered sequence of episodes, starting from a single input feature. Figure 5 shows a simulation of the network in this modality. Figure 5A reports the temporal activity of the pyramidal neurons (zp) of three layers of the network: Working Memory, “Theta generator,” and Layer 2 (the activity in Layer 1 is not shown for briefness since it is a noisy version of the same activity as in Layer 2). During the simulation, the first episode of each sequence is provided as input (see Supplementary material) to the “WM” layer for a short time (50 ms). The “WM” is able to maintain this information in memory, even when the input has ceased, and to reset when a new input is provided. The “Theta generator” layer oscillates autonomously with a theta rhythm (~ 4 Hz) and acts as a disinhibitor on the “L1” layer. The latter, together with the “L2” layer, entirely reconstructs the first episode from a single feature, and recalls all subsequent episodes in the sequence. The process occurs through oscillations with the gamma rhythm (~35 Hz) and, together with the slower rhythm transmitted by the “Theta generator,” generates the so-called theta-gamma code. “L2” is considered the output of the network. Figure 5B shows a zoomed plot of pyramidal neurons’ activity in layer “L2.” The first feature, given as input, recalls all the remaining features of the same episode, which in turn recall the first feature again with a slight delay. Thereafter, each episode recalls all the features of the next one in a highly synchronized manner, and the overall sequence repeats during each theta cycle until a new input resets the overall sequence.

#### 3.1.2 Non-orthogonal sequences (shared features)

**Figure 6:**
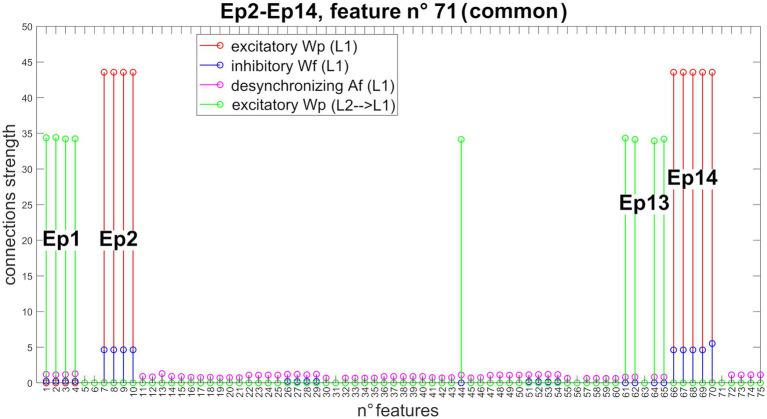
Synapses entering into Unit 71 (non-orthogonal features). In the presence of non-orthogonal episodes (see Figure 3B), feature n° 71 is shared by the second and 14th episodes. Accordingly, this feature receives excitatory and inhibitory synapses from all features of both episodes (Episode 2: features from 7 to 10, and feature 70; Episode 14: features from 66 to 70), excitatory synapses from all features of both preceding episodes (Episode 1: features from 1 to 4; and Episode 13: features 44, 61, 62, 64, and 65), and desynchronizing synapses from all other episodes. Due to the normalization mechanism, the strength of the incoming synapses to this shared feature is about half that of a not shared feature (see Figure 4 for a comparison).

Figure 6 shows the new pattern of synapses entering Unit 71 (now common between Episode 2 and Episode 14). The network can correctly store even episodes that are non-orthogonal.

**Figure 7:**
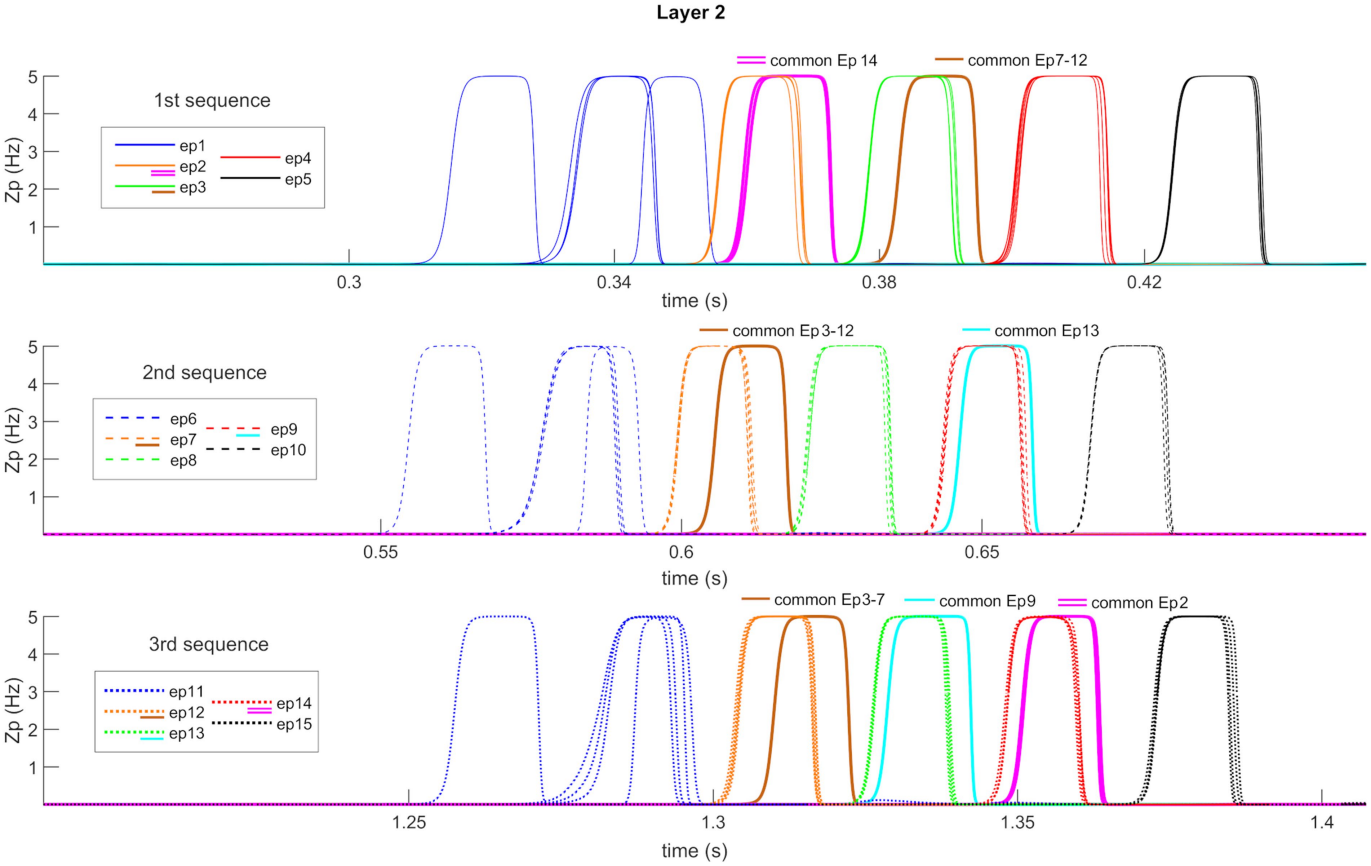
Retrieval operation mode (non-orthogonal features). Temporal activity of pyramidal neurons in layer “L2” of the network, during the *Retrieval* functioning mode, in the presence of some non-orthogonal episodes sharing common features. A simulation lasting 1.5 s is reported during which three different features, belonging to the first episode of each time sequence, are provided as input to the network. Again (see also Figure 5), the five episodes constituting each sequence are represented through a list of colors, while the sequences are distinguished through different hatching types. Moreover, additional colors (i.e., purple, brown, and light blue) represent shared features between episodes (see also the legends). Each row represents the reconstruction of a single sequence. The network can correctly evoke the three sequences, including shared features, and behaves similarly to Figure 5 about orthogonal features. Worth noting that the shared features are evoked with a little delay compared with the distinctive ones.

Figure 7 shows the temporal activity of layer “L2”s pyramidal neurons during the recovery of three non-orthogonal sequences in the *Retrieval* operation mode. Even in the presence of episodes that share one or two features (approximately from 20 to 35% of the entire episode), the network is able to correctly evoke the whole sequences in a very similar manner as in Figure 5. It should be noted, however, that features shared between multiple episodes are recalled by the network with a slight delay (compared to orthogonal features).

### 3.2 Quantitative model assessment

#### 3.2.1 Basal conditions

Model assessment with basal parameter values is reported in Table 1 for the orthogonal sequences and the non-orthogonal ones. The results can be summarized as follows: (i) The first episode of each sequence is correctly recovered in about 70% of the theta cycles. The reason is illustrated in the last line of Figure 5. The feature given as input recovers all the other features of the first episode with a slight delay; these features, in turn, excite the first feature again with a further smaller delay. In about 1/3 of cases, the superimposition between the first feature and the others does not satisfy the strict requirement we impose for object recognition. (ii) The second and third episodes of any sequence are always correctly recovered; (iii) in sporadic cases (98–99% of success), the fourth episode is not recovered; more frequently, the last (i.e., the fifth) episode is not recovered (88–89% of success). This is due to an interruption of the on phase of the theta cycle while the network is still processing the last portion of the sequence.

**Table 1:** Percentage of success in recovering the different episodes within a sequence, with basal parameter values.

		Episode 1	Episode 2	Episode 3	Episode 4	Episode 5
Orthogonal sequences	Sequence 1	77.50	100.0	100.0	100.0	90.0
	Sequence 2	57.5	100.0	100.0	100.0	90.0
	Sequence 3	75.0	100.0	100.0	97.5	87.5
	Mean	70.0	100.0	100.0	99.167	89.167
Non-orthogonal sequences	Sequence 1	77.5	100.0	100.0	100.0	90.0
	Sequence 2	60.0	100.0	100.0	97.5	90.0
	Sequence 3	77.5	100.0	100.0	95.0	82.5
	Mean	71.67	100.0	100.0	97.5	87.5

Episodes recognized in more than 66% of trials are marked in green, while episodes recognized in more than 50% than trials (but less than 66%) are marked in yellow, to give an immediate outline of network capacity.

#### 3.2.2 Sensitivity analysis of the frequency of the theta rhythm

The same analysis, concerning the non-orthogonal sequences only, has been repeated by altering the frequency of the theta rhythm produced by the “Theta generator”. This has been realized by changing the glutamatergic and slow GABAergic synapses’ time constants by the same multiplicative factor. Of course, increasing the time constants reduces the frequency, whereas their reduction makes the rhythm faster. Results are summarized in Table 2.

**Table 2:** Percentage of success in recovering the different episodes within a sequence, but different values for theta and gamma frequency.

		Episode 1	Episode 2	Episode 3	Episode 4	Episode 5
Theta = 2.67 Hz Gamma = 35 Hz	Sequence 1	73.3	100.0	96.67	100.0	100.0
	Sequence 2	56.67	83.3	100.0	100.0	100.0	Sequence 3	53.3	90.0	100.0	96.67	100.0
	Mean	61.11	91.11	98.89	98.89	100.0
	Theta = 5 Hz Gamma = 35 Hz	Sequence 1	80.0	100.0	88.0	68.0	2.0
Sequence 2		70.0	98.0	96.0	42.0	6.0	Sequence 3	76.0	100.0	96.0	46.0	6.0
Mean		75.3	99.3	93.3	52.0	4.67
Theta = 6 Hz Gamma = 35 Hz		Sequence 1	50.0	61.67	6.67	3.33	0
	Sequence 2	46.67	41.67	40.0	1.67	0	Sequence 3	65.0	51.67	23.33	0	0
	Mean	53.89	51.67	23.33	1.67	0
	Theta = 5 Hz Gamma = 45 Hz	Sequence 1	96.0	100.0	98.0	88.0	8.0
Sequence 2		92.0	100.0	100.0	78.0	20.0	Sequence 3	100.0	100.0	96.0	82.0	20.0
Mean		96.0	100.0	98.0	82.67	16.0
Theta = 4 Hz Gamma = 45 Hz		Sequence 1	95.0	100.0	97.5	97.5	97.5
	Sequence 2	92.5	100.0	100.0	100.0	100.0	Sequence 3	95.0	100.0	100.0	97.5	95.0
	Mean	94.17	100.0	99.17	98.33	97.5

The results refer to non-orthogonal sequences only. Episodes recognized in more than 66% of trials are marked in green, while episodes recognized in more than 50% than trials (but less than 66%) are marked in yellow, to give an immediate outline of network capacity.

**Figure 8:**
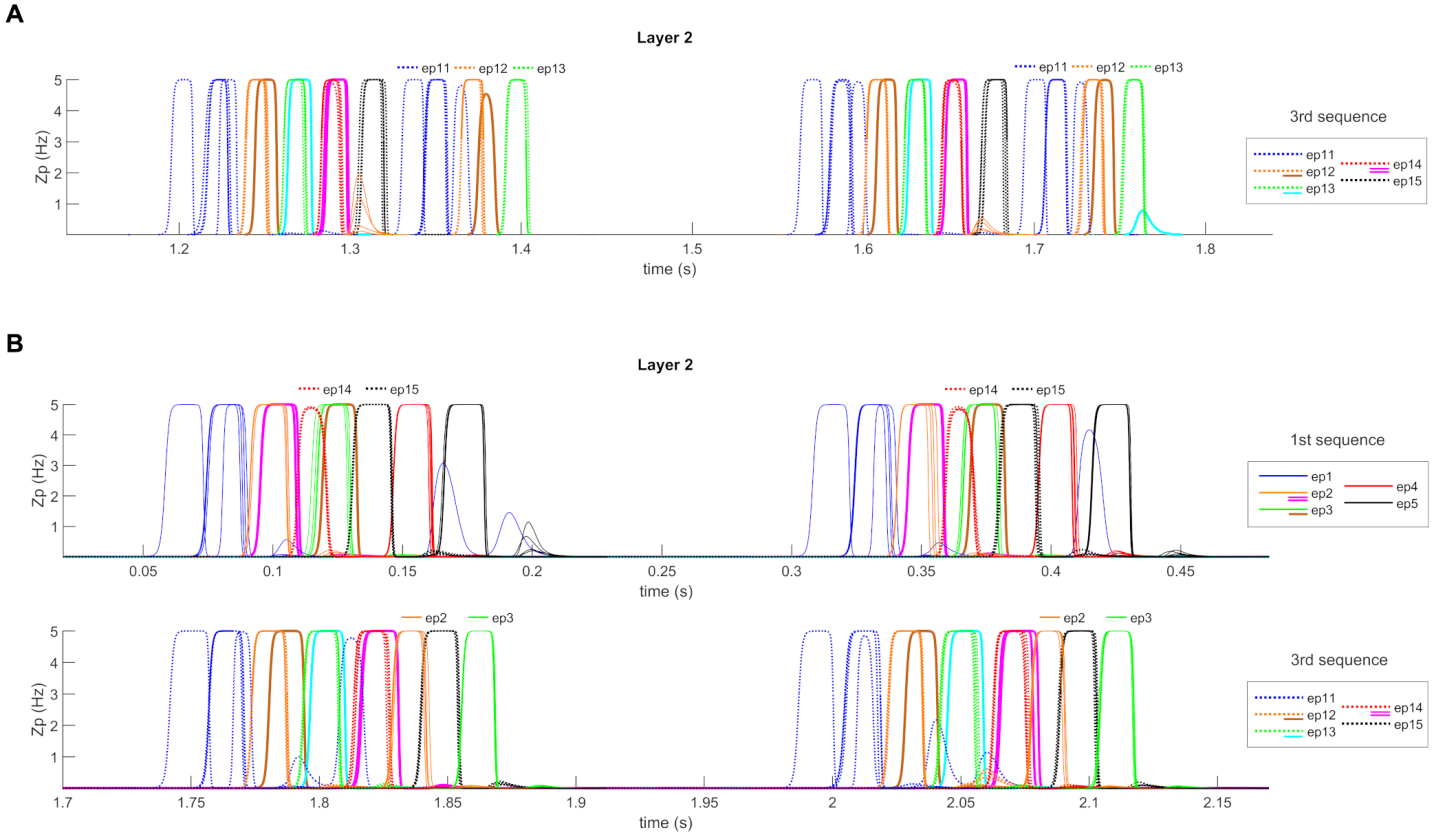
Quantitative model assessment. **(A)** Temporal activity of pyramidal neurons in layer “L2” of the network, during the *Retrieval* functioning mode, in the presence of non-orthogonal episodes and decreased theta frequency (about 2.5 Hz). A portion of the simulation is reported during which a feature belonging to the first episode of the third sequence is provided as input to the network. The five episodes are represented through a list of colors, while additional colors represent shared features between episodes (see also the legends and Figure 5). With decreased theta frequency, the capacity of the network to recover the fourth and the fifth episodes during a cycle is increased. Moreover, due to the increase period and the sustained input activity, the sequence restarts within the same cycle. Two subsequent theta cycles are shown to better highlight this behavior. **(B)** Temporal activity of pyramidal neurons in layer “L2” of the network, during the *Retrieval* functioning mode, in the presence of non-orthogonal episodes and increased gamma frequency (about 45 Hz). Two portions of one simulation are reported (in two different rows) during which a feature belonging to the first episode of the first and third sequences is provided as input to the network. With increased gamma frequency, and basal theta frequency (i.e., 4 Hz), the network behavior shows a drawback: in several cases, a spurious episode belonging to other sequences appears. For example, during the recovery of the first sequence, episodes 14 and 15 are recalled, while during the recovery of the third sequence, episodes 2 and 3 are retrieved. Also in this panel, each row shows two subsequent theta cycles.

As well expected, decreasing the theta frequency improves the capacity to recover the fourth and the fifth episodes during a cycle. Conversely, the capacity to recover the first and second episodes moderately deteriorates. Moreover, as illustrated in Figure 8A, the sequence restarts within the same cycle due to the increase in the period. Indeed, all our sequences are composed of five episodes. If the theta frequency is reduced to 2.5 Hz, eight episodes can be embedded within the same cycle. A lower theta frequency is optimal only if longer sequences must be recovered, i.e., a strict relationship exists between sequence length and theta period.

Conversely, if the theta frequency increases up to 5 Hz, only the first three episodes of each sequence can be restored quite well, whereas the fourth is restored only in 50% of cases and the last rarely. If the frequency is increased to 6 Hz, just the first two episodes are recovered with difficulty (50% of success).

#### 3.2.3 Sensitivity analysis of the frequency of the gamma rhythm

We also tested the effect of an increase in the gamma frequency, an internal property of Units in layers L1 and L2. This increase (up to 45 Hz) was realized by decreasing the time constant of fast GABAergic interneurons in both layers, and this change was tested both in case of increased theta frequency and basal conditions.

The first interesting result (Table 2) is that the network can restore up to four episodes in the same theta cycle with good accuracy, even if the theta frequency is increased from 4 to 5 Hz, provided the gamma frequency is simultaneously increased. We did not test higher gamma frequencies because these cannot be realized with the present neural mass model.

Then, we tested model behavior with a high gamma frequency (45 Hz) and basal parameter values (i.e., a theta frequency as low as 4 Hz). In this condition, network behavior seems excellent (even better than in Table 1, when the gamma frequency was 35 Hz). All five episodes within a sequence are correctly restored with a percentage of success higher than 95%. However, as illustrated in Figure 8B, there is a drawback: in several cases, a spurious episode belonging to other sequences appears mixed in with the correct episodes. During recovery of the first sequence, this occurs as to episodes 14 and 15 (which belong to the third sequence), while during recovery of the third sequence, this occurs as to episodes 2 and 3. The reason is that some episodes have shared features. In the presence of fast gamma dynamics, these shared features evoke a new episode quickly despite inhibitory connections from the correct sequence.

#### 3.2.4 Robustness analysis of Hebbian and anti-Hebbian learning rules

Finally, we tested the effect of changes in synaptic training mechanisms for all four implemented synapse types (i.e., $Wp^{L1L1}$ ; $Wf^{L1L1}$ ; $Af^{L1L1}$ ; and $Wp^{L1L2}$ ). The increase (decrease) in synaptic values was realized by either increasing (decreasing) the synaptic learning factor, decreasing (increasing) the synaptic thresholds, or increasing (decreasing) the maximum saturation value, leading to different final synaptic values. The results, summarized in Supplementary material, suggest that the network is quite robust to changes in the synaptic training mechanisms. However, we just want to emphasize how, in the case of non-orthogonal sequences, excitatory synapses cannot be increased excessively to avoid the recovery of spurious terms (due to shared features).

### 3.3 Isolation from the external world

#### 3.3.1 Imagination

**Figure 9:**
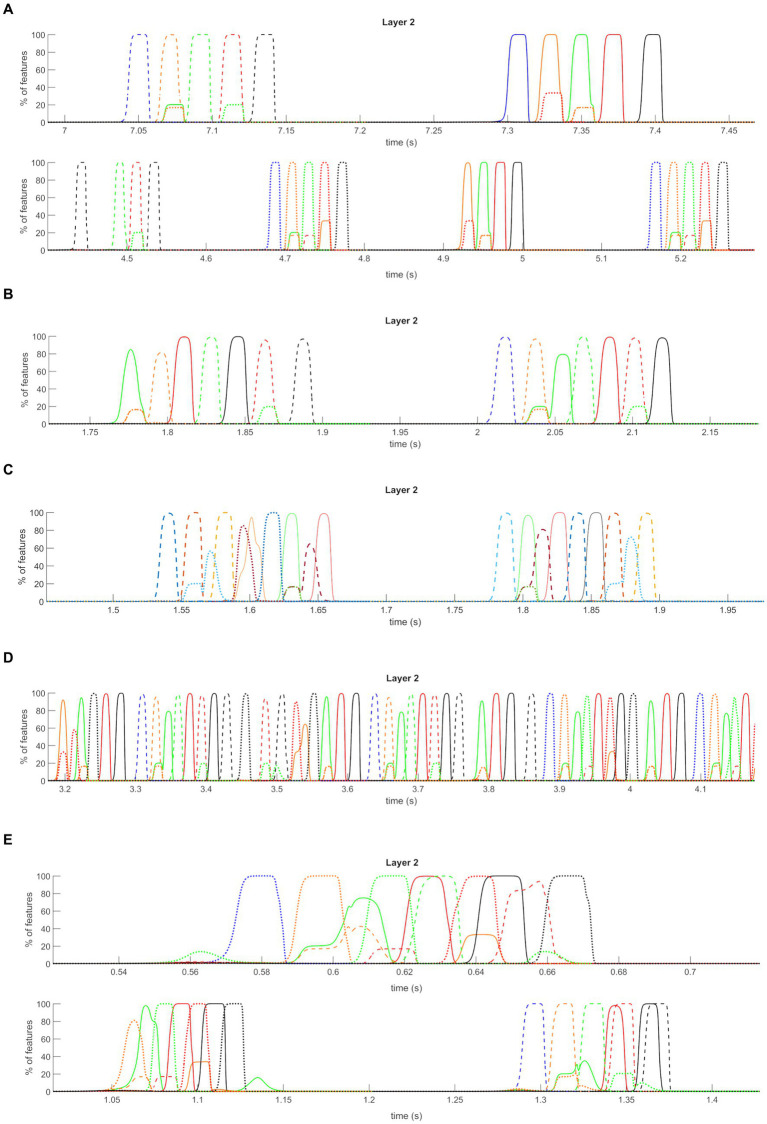
Isolation from the external world operation mode (non-orthogonal features). *Average* temporal activity of pyramidal neurons from layer “L2” of the network, during the *Isolation from the external world* mode operation, in the presence of stored non-orthogonal sequences. Only a few short segments are shown, extrapolated from a longer simulation. During this time, the “L1” layer receives a high uniform noise. Only the average activities of all features belonging to the same episode are plotted (i.e., the *y*-axis now represents the percentage of features correctly retrieved at a given instant). Three different conditions are shown. **(A)** “Imagination”: the network can autonomously and randomly recall all the sequences previously learned during the encoding phase, with the correct order of episodes in each sequence. **(B–D)** “Dreaming”: the “L1” layer receives even higher uniform noise than the “*Imagination*” mode. In addition, all the synapses are decreased by 2/3: the network autonomously and randomly creates new sequences, combining the previously trained ones and exploiting common features between episodes. The randomness of the noise is crucial in achieving different combinations from time to time. The first panel reports a simulation at basal conditions for gamma and theta rhythm, the second panel a simulation with increased gamma frequency, and the third panel a simulation with basal gamma but without modulation by theta rhythm. **(E)** “Schizophrenia”: a high uniform noise is given to the “L1” layer (as in “*Dreaming*” mode). Moreover, desynchronizing synapses $Af^{L1L1}$ are reduced to one half: the network cannot correctly recover learned sequences, and presents superimpositions of episodes, which are even more pronounced due to common features.

Figure 9A shows the behavior of the network while “L1” receives a high random noise with uniform distribution, with all synapses maintained at the same value as in the previous simulations. In these conditions, the network, without influences from the external world, casually recalls the previously stored sequences (even with shared features), which appear in random order. The authors find interesting affinities between this simulation and a state of imagination or mind-wandering.

#### 3.3.2 Dreaming

Figure 9B shows the results of a simulation when the network is still disconnected from the outside world, but this time layer “L1” receives a higher (than the “*Imagination*” mode), random noise input and a 2/3 reduction in all the synapses was assumed. Differences in neurotransmitter levels could justify the latter; for instance, a mild decrease in cholinergic tone during sleep compared with retrieval or other neurotransmitter changes. Interestingly, the network can no longer retrieve the correct sequences of episodes but instead exploits shared features to generate new “dreamlike” combinations. Specifically, common features between episodes 3 and 12 are exploited to generate two completely new sequences (initial part of the first sequence + last part of the second sequence, and initial part of the second sequence + last part of the first sequence). Furthermore, since our previous sensitivity analysis revealed that this “dreamlike” behavior can become even more evident if the gamma rhythm is accelerated, we repeated the same simulation using a gamma frequency as high as 45 Hz (Figure 9C). In this condition, all three sequences are combined generating an even more complex scenario inside the same theta wave. We find some similarities between this condition and some typical aspects of REM sleep (see “*Discussion*” for a more critical analysis).

Additionally, since an essential aspect of the present model is the possibility to modulate the theta rhythm, we repeated the last simulations by maintaining a constant (not oscillating) input from the “Theta generator” (Figure 9D). In this condition, the network simulates a non-theta state of the hippocampus, more similar to that occurring during slow-wave sleep (see again “*Discussion*”). As evident in Figure 9D, the absence of theta modulation leads to the genesis of a single long combination of the different sequences. Notably, some intermediate results between all the latter modes can be obtained by varying the value of input noise, synapse reduction, and theta on-period length (unpublished results). In other words, depending on the chosen values, the network can retrieve the stored sequences more or less correctly and consequently generate fewer or more new combinations of them (within the same simulation).

#### 3.3.3 Schizophrenia

Lastly, a third condition has been simulated by still providing high noise (as in “*Dreaming*” mode) to the network and reducing the desynchronizing synapses $Af^{L1L1}$ to a value half the original. This reduction in synapses may represent a pathological alteration connected with the weakening of some GABAergic inhibitory synapses (see “*Discussion”* section), such as in schizophrenia. Now, a different pattern can be seen. Indeed (see Figure 9E), the network still occasionally generates new combinations of sequences, but the primary drawback is that different episodes are recovered simultaneously and superimposed (i.e., features of different episodes are oscillating together, producing a distorted reality, like in “delusion”).

Interestingly, the network can still correctly recall the stored sequence of episodes if the value of synapses is reduced during the *Retrieval* operation mode. The particular behaviors described above characterize only states of isolation from the external world, like imagination or dreaming, where the randomness of noise (and, of course, the absence of environmental inputs) makes the network’s functioning more vulnerable.

## 4 Discussion

In the last decades, many results have been gathered, both in rodents and primates, as well as in humans, suggesting that the brain utilizes the precise time of neural activity to encode information. The theta-gamma code hypothesis (Lisman, 2005; Lisman and Jensen, 2013), in particular, assumes that attributes of the same item can be linked together using phase synchronization in the gamma band, while multiple items can be ordered sequentially through the phase of a modulating theta wave.

This encoding modality has been observed in several brain regions in humans, producing the idea that theta-gamma entrainment plays a broad function in cognition, subserving not only episodic and working memory but also motor command control (Spooner et al., 2020), skill acquisition (Akkad et al., 2021), and speech comprehension (Lizarazu et al., 2019). However, the most significant results have been observed in the hippocampus (Colgin et al., 2009; Mysin and Shubina, 2022), stressing the role of this mechanism in chronological episodic memory, i.e., the sequential organization of autobiographical episodes that unfold over time.

Despite the numerous neurocomputational models, which describe individual components of the hippocampus, the problem of how the theta-gamma code is organized at a mesoscopic level involving populations of neurons and how this mechanism reflects memory encoding can still benefit from a new synthetic perspective. In this regard, we use an approach based on neural masses, which provide information at a large scale, complementing the information provided by more detailed mechanistic models at a single neuron. In particular, models at a mesoscopic level can be beneficial in summarizing the main principles of brain organization, pointing out possible relationships among areas or regions, and clarifying the role of connectivity patterns. Furthermore, due to their simplicity and synthetic attitude, they can help explain the impact of parameter changes and drive the development of new general ideas on the relationship between brain structure and cognition. On the other hand, detailed neuron models are indispensable to understanding mechanisms at the membrane, ionic channels, and synapses, studying pharmacological and neurotransmitter effects, and posing physiological constraints on more abstract models.

### 4.1 Limits of the previous model

In recent years, we developed a model in which working and long-term memories are strictly interrelated, as suggested in several recent studies (Renoult et al., 2019; Köster and Gruber, 2022). A fundamental aspect of the previous model is that both the theta and gamma rhythms are not inherent properties of the network but are generated after training through plastic auto-associative synapses: the theta rhythm emerges as a consequence of excitatory Hebbian synapses trained to recover items in memory, which excite pyramidal neurons, subsequently inhibited by slow GABAergic interneurons; the gamma rhythm is produced in a downstream layer as a consequence of Hebbian and anti-Hebbian synapses which target fast GABA-ergic inhibitory interneurons.

As discussed in the “*Introduction*,” recent data show the presence of non-theta states in the hippocampus; hence, a question arises: how can the theta rhythm be suppressed or modulated in the previous model? To abolish the theta-rhythm, pyramidal neurons must remain excited despite the inhibition from slow GABAergic interneurons. This requires a higher sustained global excitation.

In the previous paper, the model was used with two different modalities. In the first, named “semantic working memory,” the theta rhythm was suppressed using high values for the inputs, and the network could reconstruct and desynchronize up to nine objects simultaneously held in memory in a non-theta state. However, in a second modality, named “sequential order modality,” the network exploited a theta-gamma code to order items within a sequence. This condition, the same simulated in the present work, is observed in the hippocampus during active exploratory behavior, cognitive tasks requiring attention, locomotion, and rapid eye movement (REM) sleep (Mysin and Shubina, 2023). However, in this modality, the previous model cannot explain several phenomena well documented in the recent literature. First, the theta rhythm is rapidly suppressed during quiet wakefulness and slow wave sleep (SWS) when the hippocampus is isolated, a condition that we could not simulate with the previous internal mechanism for theta rhythm generation. Second, it is difficult to modulate the frequency of the theta rhythm (which, conversely, changes in various behavioral states, for instance, when a rodent is running inside a maze; Hinman et al., 2016). Third, the model cannot explain how the theta rhythm can be affected by pacing MS cells or by MS lesions. Finally, several authors hypothesized that different phases of the theta rhythm can be used for memory encoding and retrieval, respectively. This behavior has been ascribed to rhythmic acetylcholine changes (Hasselmo et al., 1995; Hasselmo, 2006). High acetylcholine levels can depress feedback synapses, thus avoiding interference between old and new memory traces. In contrast, low acetylcholine levels can favor activity spreading along feedback synapses, thus restoring memories through attractor dynamics. All these aspects suggest the presence of an external pacemaker for theta (and also for acetylcholine) and of a gamma rhythm able to adapt to theta changes.

In conclusion, the previous model is satisfactory when used as a semantic memory to segment different items without a theta rhythm. Conversely, it is not robust or flexible enough to simulate conditions associated with a sequential temporal code, mainly to explain the transition from theta to a non-theta condition, MS pacing or MS lesions, and rhythmic modulation of the encoding phase. Hence, we decided to modify the model, considering an external theta pacing.

### 4.2 Present model

In the present work, we tested an alternative model based on more straightforward assumptions, considering that the gamma rhythm is generated internally at each computational Unit due to local not-trainable feedback connections between pyramidal and fast-GABAergic populations (hence, parameters internal to each Unit have been modified compared with the previous work). Moreover, the theta rhythm is produced by an external oscillator, therefore exploiting another Unit with slower internal dynamics. Finally, Hebbian and anti-Hebbian auto-associative synapses are still used to reconstruct episodes from an external cue, but they are present in a single layer. Hence, the structure of the current model is much simpler than the previous structure. Nevertheless, it works satisfactorily to reconstruct an event characterized by different episodes that unfold in time with a given sequential order; the isolated and noisy network can also simulate other mental conditions, such as imagination or dreaming.

In the following, the main assumptions of the model are first discussed, emphasizing possible neurophysiological support concerning the hippocampal regions. Next, points that are still hypothetical will be critically underlined. Finally, some testable predictions will be enumerated, able to discriminate among the different models and furnish elements to validate or reject hypotheses, and lines for future improvements pointed out.

### 4.3 The structure of the model

The model exhibits a three-layer structure in which the same Units, coding for individual features, are identically replicated in each layer. Of course, this is a simplification of an actual multi-layer network. In multi-layer networks used in deep learning (Aggarwal, 2018), the featural representation becomes progressively more general and abstract when moving from an upstream to a downstream layer. A similar computation is inspired by the visual processing pathway. However, we are not aware of whether a change in representation also occurs along the hippocampus (from CA3 to CA1). Hence, we retained a straightforward structure with constant feature coding. A different, more complex structure of the present network, in which the feature representation changes from one layer to the next, can be the subject of future studies.

In the typical *Retrieval* modality, the layer denoted “WM” receives the external input and implements a simple working memory to maintain the input and resets it as soon as a new input arrives. A possible location for this layer is the prefrontal cortex and/or the entorhinal cortex (Bolkan et al., 2017; Eichenbaum, 2017b). We included a positive self-loop for the pyramidal population to maintain information in the “WM” layer, realized through excitatory glutamatergic synapses. This loop is then interrupted at each new input to reset the memory content. Results in the literature suggest that a similar self-loop can be realized via reciprocal connectivity linking the prefrontal cortex, the thalamus, and the basal ganglia (Bolkan et al., 2017; Nir-Cohen et al., 2020). A more accurate modeling of this loop can be furnished in future work.

Layer “L1” implements a classic auto-associative memory, which can restore a complete episode from a single feature (provided this feature is not shared with other episodes). To this end, three different types of lateral synapses are potentiated during a training epoch (simulating an encoding phase), starting from an initial null value (see below). It is worth noting that in the previous model, excitatory and inhibitory synapses were separately trained in different layers (to realize the theta and gamma rhythms distinctly). Conversely, all lateral synapses in the present model are within layer “L1.” This layer likely mimics the region CA3 of the hippocampus, where recurrent synapses with Hebbian potentiation are well known (Treves and Rolls, 1994; Hasselmo et al., 1995). An essential suggestion of the model is that all three types of synapses ( $Wp^{L1L1}$ , $Wf^{L1L1}$ , and $Af^{L1L1}$ ) are required to achieve a correct behavior. Briefly, excitatory pyramidal-pyramidal glutamatergic synapses ( $Wp^{L1L1}$ ) are necessary to excite silent Units within a given episode starting from partial information; bi-synaptic inhibition (pyramidal-fast inhibitory-pyramidal, through glutamatergic synapses $Wf^{L1L1}$ ) are essential to avoid an excessive excitation within the network, thus preserving the gamma rhythm, and to favor synchronization among features within a same episode. Several recent studies, both experimental (Hasenstaub et al., 2005; Salkoff et al., 2015) and theoretical (Bartos et al., 2002; Vida et al., 2006) emphasize the role of inhibitory connections to favor synchronism among neural oscillators. Accordingly, if $Wf^{L1L1}$ synapses are reduced, poor gamma synchronization results. Finally, very rapid synapses ( $Af^{L1L1}$ , targeting fast inhibitory interneurons, with synapse dynamics smaller than 1 ms) are necessary to desynchronize items belonging to different episodes so that just one episode (with all its features synchronized) spreads out at each time. As said before, the presence of Hebbian glutamatergic synapses is well documented in CA3. Some authors recently (Schmitz et al., 2001; Molchanova et al., 2016) demonstrated the presence of gap junctions between CA3 cells, providing a mechanism for high-speed communications and synchronization. Our model suggests a role for gap junctions between pyramidal and fast interneurons to help desynchronization and also indicates that these synapses (or, alternatively, very fast AMPA synapses) should be anti-Hebbian. This point represents a subject for future study and future testable predictions (see also the section “Testable predictions” below).

In our model, “L1” is ordinarily silent and is disinhibited by activity from an external Unit, oscillating with a theta period. Although recent data suggest that a theta rhythm can also be internally generated within hippocampal circuits (Cataldi and Vigliotti, 2018), as done in our previous model, or modulated by other hippocampal structures such as the entorhinal cortex (Schlesiger et al., 2015), a more common idea is that the theta rhythm originates from an external structure: this may be the septum (Salib et al., 2019), or a resonance within the Papez circuit (including the mammillary bodies, the mammillothalamic tract, and the anterior thalamic nuclei; Vertes et al., 2001; Dillingham et al., 2021). In particular, Salib et al. (2019) suggest that the septum can act by disinhibiting the hippocampus with a mechanism analogous to the one included in our model. An improved version of the current model may take into account several aspects not implemented here, such as mimicking theta rhythm modulations experimentally detected during different locomotor speeds (Bender et al., 2015), respiratory frequencies (Tsanov et al., 2014), or emotional states (Colgin, 2013).

Finally, we can hypothesize that layer “L2” is located in the region CA1 of the hippocampus. It is well known that CA1 cells receive spatially organized projections from CA3 cells (the so-called Schaffer collaterals) (Hongo et al., 2015). Moreover, several results underline the importance of CA1 for temporal coding and organization (Hoge and Kesner, 2007; Mankin et al., 2012), indirectly supporting our network structure. While the restoration of individual episodes in our model can be entirely accomplished within “L1,” a temporal organization of individual episodes that unfold in time depends on the interaction between “L1” and “L2” and, mainly, on the formation of feedback excitatory synapses from “L2” to “L1.”

Although feedback from CA1 to CA3 needs to be more documented compared with feedforward Schaffer collaterals, some recent data support its possible involvement in hippocampus dynamics. For example, connections from CA1 to CA3 can be realized via the participation of the entorhinal cortex (see Figure 2 in Eichenbaum, 2017b and also Craig and Commins, 2005). In addition, Sandler et al. (2015) using Granger connectivity, observed the presence of a causal relationship from CA1 to CA3.

It is worth noting that, in the present model version, we do not need plastic lateral synapses within “L2” since object reconstruction entirely occurs within “L1,” i.e., this model is much simpler than the previous. However, we know that lateral plastic synapses have been experimentally observed among CA1 cells (Tetteh et al., 2019) and that MS can modulate theta-gamma coupling in the CA1 nucleus as well (Király et al., 2023). Of course, we do not claim that these synapses and modulation do not exist or have no role at all: they are not essential for the present model’s purposes but might come into play to perform more complex computations not considered here.

Finally, although the structure of the current model can be compared with that of the hippocampus, theta-gamma entrainment has been observed in several other brain regions, too, and seems to represent a more general computation code (Lisman and Jensen, 2013). Hence, similar network principles, such as those developed in the present work, could help simulate processing in other brain structures.

### 4.4 Assumptions on model training

During our *Training*, layer “L1” is disconnected from “WM,” layer “L2” is disconnected from “L1” (i.e., the feedforward synapses have no role), and both “L1” and “L2” directly receive the input from the cortex. This particular configuration can be justified by alterations in acetylcholine (*Ach*) concentration occurring during active wakefulness and sustained attention, as suggested by Hasselmo (2006) and Decker and Duncan (2020). In these conditions, Ach levels increase, making the hippocampus ready to encode new memory traces. In fact, a peak of Ach may inhibit memory reactivation within CA3 by reducing the strength of lateral synapses (Hasselmo et al., 1995); moreover, it suppresses excitatory transmission from CA3 to CA1 (Hasselmo and Schnell, 1994) and strengthens the cortical input to CA3 and CA1. Furthermore, acetylcholine facilitates long-term potentiation between active neurons (Blitzer et al., 1990). This situation is ideal for encoding new episodes within hippocampal synapses and reflects what has been implemented in our *Training*.

A strong assumption concerns how episodes are transmitted to the “L1” and “L2” layers during *Training*: we assumed that the preceding episode of a sequence is sent to “L2” while the subsequent episode is simultaneously transmitted to “L1.” This allows the formation of plastic synapses from “L2” to “L1” to recover the entire sequence. Of course, this mechanism presumes the existence of a buffer, which maintains episodes and transmits them with a given delay. At present, we do not have physiological support for this peculiar mechanism. The necessity of a buffer (i.e., a top-down stack) has been previously hypothesized by other researchers working on temporal episodic memory, starting from the pioneering work by Jensen and Lisman (1996). In more recent work, Lisman et al. (2005) assumed delayed feedback from CA3 to the dentate gyrus to realize this stack. In their paper (Lisman et al., 2005), this idea was formulated since “there are no connections from CA1 back to the dentate or CA3.” However, as mentioned above, more recent data suggest the existence of a bidirectional pathway between CA1 and the lateral entorhinal cortex (and from there to CA3) (Craig and Commins, 2005; Eichenbaum, 2017b) while a Granger causality has been observed from CA1 to CA3 (Sandler et al., 2015).

### 4.5 Model functioning

After encoding information in plastic synapses, the network’s behavior has been simulated in different modalities. In the first, which mimics regular memory retrieval, the network can restore an entire sequence of episodes starting from a distinctive feature. In this condition, the “L1” layer receives the external input from an upstream working memory circuit and is disinhibited by an external Unit oscillating with the theta rhythm. This allows the implementation of a robust theta-gamma code, i.e., all features of a single episode are synchronized within a single gamma period. A sequence of episodes is reconstructed and replicated during the on phase of each theta cycle. Remarkably, we used just five episodes per each sequence since this is the maximum number of our gamma cycles that can be nested within the on phase of our theta period; if longer sequences are memorized (unpublished simulations), only the first five episodes are restored within each theta period; moreover, if the external input is shifted, a typical precession phenomenon can be simulated. However, as shown in the sensitivity analysis, a slower theta might allow restoration of longer sequences.

An original fundamental aspect of our work is that the network can be run in other modalities (“Imagination,” “Dreaming,” or “Schizophrenia”): “L1” is isolated from the Working Memory, and the network receives only high internal noise. In the modality named “Imagination,” we maintained the same values for the synapses as in the regular *Retrieval* modality, only increasing the noise level. Interestingly, the “Imagination” modality can correspond to conditions characterized by low acetylcholine levels, which reduces the input to CA3 and can promote pattern completion and memory reply (Hasselmo, 2006; Decker and Duncan, 2020). A significant result is that, in this condition, the network can autonomously and randomly recall the previously stored sequences, i.e., it replays experience independently of any external input.

Even more interesting, in a different condition that we named “Dreaming,” characterized by even stronger noise and a 2/3 reduction in all synapses’ strength, the network randomly recombines previously stored sequences, thus creating new original sequences. This possibility depends on features that are not distinctive but shared among different episodes, i.e., on a lack of orthogonality. The latter aspect appears essential to generate such “creative” behavior. Moreover, this “creative remembering” can be further favored by an increase in gamma frequency. The existence of a phase with higher noise and reduced synapses is hypothetical. Still, it can be justified by thinking of an intermediate acetylcholine level (i.e., a level moderately more elevated than the one assumed during the imagination or retrieval phase but still significantly smaller than in the encoding phase). Indeed, an increase in acetylcholine concentration reduces the strength of the synapses both within CA3 and between CA3 and CA1. Other neurotransmitter changes can also affect the network, causing a similar behavior. This aspect, of course, requires further study. Another possibility is a downscaling of synapses hypothesized by some authors during sleep (Tononi and Cirelli, 2006).

Nevertheless, it is well known that during REM sleep, the acetylcholine concentration reaches high levels (comparable to those observed during awake, attentive states see Hasselmo, 1999), a condition that facilitates information encoding. Hence, simulation of REM sleep requires a more complex scenario, which can alternate encoding and retrieval. On the contrary, the cholinergic tone is significantly reduced during slow-wave sleep (SWS). Actually, during SWS, the theta rhythm is replaced by a much slower rhythm in the cortex, while CA3 and CA1 pyramidal cells do not show OFF states and are not bistable (Isomura et al., 2006). For this reason, we repeated our simulations in the “Dreaming” conditions by eliminating the theta rhythm and maintaining a constant disinhibition to the layer “L1” (see Figure 8D). In this condition, the network can generate longer sequences by combining the previous ones on the basis of shared features. We trained the network with only three sequences having some common features. Of course, more complex and “creative” behavior can be obtained using a greater number of stored sequences.

We are aware that during SWS replay occurs on a faster time scale and with the production of spindles and sharp-wave ripples, more complex rhythmic phenomena than those considered in the present work (Brodt et al., 2023). This could be investigated using a more detailed model (see the section “Future lines” below).

Some authors (Diekelmann and Born, 2010) hypothesized that SWS and REM sleep have complementary functions: SWS should be associated with system consolidation in which hippocampal replay leads to re-activation in the cortex. Conversely, the ensuing REM sleep would promote synapse strengthening. Future work can focus on realizing an integrated model of hippocampus + cortex to study the complementary role of SWS and REM sleep.

### 4.6 Testable predictions

Some testable predictions concern the general structure of the network. Others can discriminate between the present and previous models (Ursino et al., 2023). Finally, some involve specific parameter alterations.

Testable aspects of the general model structure involve high-speed connections (maybe gap-junctions or AMPA fast synapses) within CA3 and plastic Hebbian synapses from CA1 to CA3 (maybe through the lateral entorhinal cortex). Experiments can be devised to unmask the role of these connections and their plastic changes.

Other testable predictions concern the effect of synapse strength on the global network behavior. Experiments with different levels of acetylcholine or using neurotransmitters that alter synapses can be performed and compared with model behavior in similar conditions.

Predictions able to discriminate the present model from the previous concern the genesis of the theta and gamma rhythm: the previous model suggested that theta and gamma rhythms arise after training. In contrast, in the present model, they are intrinsic to hippocampal cells (gamma) and externally produced (theta). Furthermore, in the current model, lateral plastic synapses are essential only in “L1” (CA3) and are directed to pyramidal and inhibitory interneurons. Conversely, the previous model confined excitatory and inhibitory synapses in different layers.

The effect of some parameter alterations (for instance, the activity of GABAergic neurons, of the theta Unit, feedforward connection strength, etc.) can be simulated in the model, and results can be compared with experimental manipulation of the same parameters (either obtained via drugs or surgical procedures). In particular, the analysis of feedback connections from CA1 to CA3 is a major point for future model testing.

Another important point to be stressed is that the presence of a moderate synapse reduction during dreaming is a strong prediction of the model: in the presence of decreased recurrent synapses the memorized sequences become less “stable” or weaker, and so it becomes easier to branch from one sequence to another in a random and creative way by exploiting the presence of shared features.

A final powerful prediction of the model concerns the pivotal role of noise during imagination and dreaming states: a high level of noise in the neural populations seems an essential element for these mental states. This aspect (along with synaptic reduction during dreaming) may be the subject of fascinating investigations in the future.

### 4.7 Future lines

First, the present network can be integrated with a network representing the cortex’s activity, including semantic aspects. This integrated hippocampal-cortex model can then analyze memory consolidation, i.e., the process that transforms new and initially labile memories and integrates them into a network of pre-existing long-term memories. The common idea is that episodes are only temporarily encoded in the hippocampus and then transmitted to a long-term store in the cortex. Here, episodes can also be integrated with other kinds of memory, like the semantic one (Ursino et al., 2015, 2018; Ursino and Pirazzini, 2023). It is worth-noting that this theta-acetylcholine modulation could be easily implemented within the present model in future work, whereas it is of difficult implementation with the previous model.

Second, the model can be enriched with a more explicit description of the acetylcholine concentration changes and their effect on synapses. As summarized above, this can be of value to better understand the complementary role of SWS and REM sleep. Moreover, some authors hypothesized that phasic changes in acetylcholine concentration in the awake state could regulate the theta rhythm (Gu and Yakel, 2022). Acetylcholine levels would be low during the on phase of the theta period and high during the off phase. Accordingly, the on phase would be characterized by the replay of previously stored episodes (as in our *Retrieval* modality) exploiting the theta-gamma code. At the same time, the off period would be ideal for encoding new information (as in our *Training* modality).

An improved model (for instance, using an accurate description of fast-spiking activity in the thalamus, as in Cona et al. (2014)) could also produce spindles and fast-wave ripples, which are essential to improve the simulation of replay during SWS.

Finally, the present model can also study how parameter alterations affect memory processes (encoding and retrieval), thus shading light into neurological deficits. An example concerning fast inhibitory synapses has already been presented in Figure 9E. For example, several studies suggest a deficiency of fast GABAergic interneurons in schizophrenia (Shaw et al., 2020), which weakens their inhibitory control of pyramidal cells and causes a reduction of power in the gamma band. Other studies suggest a relationship between altered theta-gamma coupling and working memory deficits in individuals with Alzheimer’s Disease or mild cognitive impairment (Goodman et al., 2018). A compromised precision of theta-gamma coupling has also been postulated as a possible cause for the decline in associative memory in old age (Karlsson et al., 2022). The availability of synthetic neurocomputational models of theta-gamma role in memory can represent an innovative and promising tool for helping the mechanistic understanding of these fundamental clinical problems.

## Data availability statement

The raw data supporting the conclusions of this article will be made available by the authors, without undue reservation.

## Author contributions

GP: Data curation, Formal Analysis, Methodology, Software, Visualization, Writing – original draft, Writing – review & editing. MU: Conceptualization, Formal Analysis, Funding acquisition, Methodology, Software, Supervision, Visualization, Writing – original draft, Writing – review & editing.
