# Supplementary material for: Modeling the contribution of theta-gamma coupling to sequential memory, imagination, and dreaming
Source: Front Neural Circuits. 2024 Jun 14;18:1326609. doi: 10.3389/fncir.2024.1326609 (PMC11211613; doi:10.3389/fncir.2024.1326609)
Supplement: Supplementary file 1 [file Data_Sheet_1.DOCX]

**Supplementary Materials**

A quantitative presentation of the model is given here, with its equations, training rules and all related parameters.

***Synapses*** – All synapses in the model are described by the following second order differential equation:

$$\begin{aligned} \frac{d^{2}y_{n}\left( t \right)}{dt^{2}}= \frac{G_{n}}{\tau_{n}}z_{n}\left( t \right)- \frac{2}{\tau_{n}}\frac{dy_{n}\left( t \right)}{dt}- \frac{y_{n}\left( t \right)}{\tau_{n}^{2}} \#(A1) \end{aligned}$$

where $G_{n}$ is the gain, $\tau_{n}$ is the time constant and *z_n_* is the input to the synapse, i.e. the presynaptic spike density. The subscript *n* is a generic one; it stands for either *p*, *e*, *s*, or *f*, depending on the neural population the equation is referring to: *p* for pyramidal neurons, *e* for excitatory interneurons, *s* for slow inhibitory interneurons, *f* for fast inhibitory interneurons. All second order differential equations of type *(A1)* are equivalent to the two first order differential equations that follow.

$$\begin{aligned} \left\{ \begin{aligned} \frac{dy_{n}\left( t \right)}{dt}=x_{n}\left( t \right) \\ \frac{dx_{n}\left( t \right)}{dt}= \frac{G_{n}}{\tau_{n}}z_{n}\left( t \right)- \frac{2}{\tau_{n}}x_{n}\left( t \right)- \frac{y_{n}\left( t \right)}{\tau_{n}^{2}} \end{aligned} \right.\#\left( A2 \right) \end{aligned}$$

***Model of a single computational Unit***– For each neuronal population, we first computed the mean membrane potential *v(t)*, which is influenced by synaptic connections. Then we computed the average firing rate of the population, *z(t)*, through a sigmoidal activation function, *S(v(t))*. Finally, a normalized post-synaptic potential, *y(t)*, can be computed using the equations *(A2)*; the latter must be multiplied by the synaptic weight to determine the actual contribution to the post-synaptic membrane potential.

Both pyramidal neurons and fast inhibitory interneurons can receive an external input – labeled *u_p_* and *u_f_*, respectively. These are random variables with normal distribution, mean value *m_p_* (or *m_f_*) and standard deviation *σ_p_* (or *σ_f_*). Both reach the target population through an excitatory synapse. In the case of *u_p_*, we implemented a common mathematical procedure to reduce the number of differential equations (and therefore, the number of state variables). Specifically, we processed *u_p_* through the excitatory synapse that goes from excitatory interneurons to pyramidal neurons (see Eqs. *(A4)* below), instead of processing the input separately. The other external input, *u_f_*, reaches its target population through a dedicated synapse (Eqs. *(A6)*).

The membrane potential of pyramidal neurons and fast inhibitory interneurons is also influenced by long-range synapses, which connect different Units. Such contributions are labelled *E*(*t*) and *I*(*t*) and will be discussed in the next paragraph.

*Equations for all populations.*

Pyramidal neurons:

$$\begin{aligned} \left\{ \begin{aligned} \begin{aligned} v_{p}\left( t \right)=C_{pe}y_{e}\left( t \right) + C_{pp}y_{p}\left( t \right)- C_{ps}y_{s}\left( t \right)- C_{pf}y_{f}\left( t \right)+E(t) \\ S\left( v_{p}(t) \right)=z_{p}\left( t \right)= \frac{2e_{0}}{1+ e^{r(s_{0} - v_{p})}} \\ \frac{dy_{p}\left( t \right)}{dt}=x_{p}\left( t \right) \end{aligned} \\ \frac{dx_{p}\left( t \right)}{dt}= \frac{G_{e}}{\tau_{e}}z_{p}\left( t \right)- \frac{2}{\tau_{e}}x_{p}\left( t \right)- \frac{y_{p}\left( t \right)}{\tau_{e}^{2}} \end{aligned} \right. \#\left( A3 \right) \end{aligned}$$

It is worth noting that the self-loop ‘*Cpp*’ of pyramidal neurons has been used only in the “WM” layer, and is set to zero in the other layers (“L1” and “L2”) and in the “Theta generator”.

Excitatory interneurons:

$$\begin{aligned} \left\{ \begin{aligned} \begin{aligned} v_{e}(t)=C_{ep}y_{p}\left( t \right) \\ {S\left( v_{e}(t) \right)=z}_{e}\left( t \right)= \frac{2e_{0}}{1+ e^{r(s_{0} - v_{e})}} \\ \frac{dy_{e}\left( t \right)}{dt}=x_{e}\left( t \right) \end{aligned} \\ \frac{dx_{e}\left( t \right)}{dt}= \frac{G_{e}}{\tau_{e}}\left( z_{e}\left( t \right)+ \frac{u_{p}}{C_{pe}} \right)- \frac{2}{\tau_{e}}x_{e}\left( t \right)- \frac{y_{e}\left( t \right)}{\tau_{e}^{2}} \end{aligned} \right. \#\left( A4 \right) \end{aligned}$$

Slow inhibitory interneurons:

$$\begin{aligned} \left\{ \begin{aligned} \begin{aligned} v_{s}(t)=C_{sp}y_{p}(t) \\ S\left( v_{s}(t) \right)=z_{s}\left( t \right)= \frac{2e_{0}}{1+ e^{r(s_{0} - v_{s})}} \\ \frac{dy_{s}\left( t \right)}{dt}=x_{s}\left( t \right) \end{aligned} \\ \frac{dx_{s}\left( t \right)}{dt}= \frac{G_{s}}{\tau_{s}}z_{s}\left( t \right)- \frac{2}{\tau_{s}}x_{s}\left( t \right)- \frac{y_{s}\left( t \right)}{\tau_{s}^{2}} \end{aligned} \right. \#\left( A5 \right) \end{aligned}$$

Fast inhibitory interneurons:

$$\begin{aligned} \left\{ \begin{aligned} \begin{aligned} v_{f}\left( t \right)=C_{fp}y_{p}\left( t \right)- C_{fs}y_{s}\left( t \right)- C_{ff}y_{f}\left( t \right)+y_{l}\left( t \right)+I(t) \\ S\left( v_{f}(t) \right)=z_{f}\left( t \right)= \frac{2e_{0}}{1+ e^{r(s_{0} - v_{f}(t))}} \\ \frac{dy_{f}\left( t \right)}{dt}=x_{f}\left( t \right) \end{aligned} \\ \frac{dx_{f}\left( t \right)}{dt}= \frac{G_{f}}{\tau_{f}}z_{f}\left( t \right)- \frac{2}{\tau_{f}}x_{f}\left( t \right)- \frac{y_{f}\left( t \right)}{\tau_{f}^{2}} \\ \frac{dy_{l}\left( t \right)}{dt}=x_{l}\left( t \right) \\ \frac{dx_{l}\left( t \right)}{dt}= \frac{G_{e}}{\tau_{e}}{(u}_{f}\left( t \right))- \frac{2}{\tau_{e}}x_{l}\left( t \right)- \frac{y_{l}\left( t \right)}{\tau_{e}^{2}} \end{aligned} \right. \#\left( A6 \right) \end{aligned}$$

where the subscript *l* is used to represent the quantities in the additional synapse, introduced to describe the effect of the input *u_f_* via glutamatergic dynamics. All related parameters’ values are listed in the tables below. Note that the variance of the noise is divided by the integration step, *dt*. In this way, we obtain a white noise with power density equal to *σ*^2^ *dt* = 5.

*Table A1: Parameters describing the dynamics of the populations within “WM” Units*

| **Synapses** | | | | **Fuction S(*v(t)*)** | |
| --- | --- | --- | --- | --- | --- |
| *G_e_* (mV) | 5.17 | *τ_e_* (ms) | 8 | *e_0_* (Hz) | 2.5 |
| *G_s_* (mV) | 4.45 | *τ_s_* (ms) | 33.4 | *r* (mV^-1^) | 0.56 |
| *G_f_* (mV) | 57.1 | *τ_f_* (ms) | 2.5 | *s_0_* (mV) | 12 |
| **Inputs** | | **Intra-unit connections** | | *C_pp_* | 2000 |
| *m_p_* (Hz) | *Retrieval*: 5000 (detected feature) or 0 (undetected features) | *C_ep_* | 54 | *C_fp_* | 108 |
| *m_f_* (Hz) | 0 | *C_pe_* | 54 | *C_fs_* | 27 |
| *σ_p_^2^* (s^-2^) | 5/dt | *C_sp_* | 54 | *C_pf_* | 300 |
| *σ_f_^2^* (s^-2^) | 5/dt | *C_ps_* | 67.5 | *C_ff_* | 10 |

*Table A2: Parameters describing the dynamics of the populations within “Theta generator” Unit*

| **Synapses** | | | | **Fuction S(*v(t)*)** | |
| --- | --- | --- | --- | --- | --- |
| *G_e_* (mV) | 5.17 | *τ_e_* (ms) | 15.7 | *e_0_* (Hz) | 2.5 |
| *G_s_* (mV) | 4.45 | *τ_s_* (ms) | 39.2 | *r* (mV^-1^) | 0.56 |
| *G_f_* (mV) | 57.1 | *τ_f_* (ms) | 3.9 | *s_0_* (mV) | 12 |
| **Inputs** | | **Intra-unit connections** | |  |  |
| *m_p_* (Hz) | *Retrieval* and *Isolation*: 500 | *C_ep_* | 54 | *C_fp_* | 27 |
| *m_f_* (Hz) | 0 | *C_pe_* | 54 | *C_fs_* | 15 |
| *σ_p_^2^* (s^-2^) | 5/dt | *C_sp_* | 54 | *C_pf_* | 300 |
| *σ_f_^2^* (s^-2^) | 5/dt | *C_ps_* | 67.5 | *C_ff_* | 10 |

*Table A3: Parameters describing the dynamics of the populations within “L1” and “L2” Units*

| **Synapses** | | | | **Fuction S(*v(t)*)** | |
| --- | --- | --- | --- | --- | --- |
| *G_e_* (mV) | 5.17 | *τ_e_* (ms) | 8 | *e_0_* (Hz) | 2.5 |
| *G_s_* (mV) | 4.45 | *τ_s_* (ms) | 33.4 | *r* (mV^-1^) | 0.56 |
| *G_f_* (mV) | 57.1 | *τ_f_* (ms) | 2.5 | *s_0_* (mV) | 12 |
| **Inputs** | | **Intra-unit connections** | |  |  |
| *m_p_* (Hz) | - See below (*Training*)  - 0 (*Retrieval*)  - Random with uniform distribution (*Isolation*). Specifically: i) Imagination:  150 - 300  ii) Dreaming:  250 - 500  iii) Schizophrenia:  250 - 500 | *C_ep_* | 54 | *C_fp_* | 108 |
| *m_f_* (Hz) | - See below (*Training*)  - 0 (*Retrieval* and *Isolation*) | *C_pe_* | 54 | *C_fs_* | 27 |
| *σ_p_^2^* (s^-2^) | 5/dt | *C_sp_* | 54 | *C_pf_* | 300 |
| *σ_f_^2^* (s^-2^) | 5/dt | *C_ps_* | 67.5 | *C_ff_* | 10 |

***Long rage connections*** – Long-range synapses connect two different Units, either belonging to the same layer or to different layers. For a more detailed qualitative description of the synaptic architecture we refer to the main text. In the following, we will use subscripts (*i* or *j*) to denote the position of a Unit within a layer, and superscripts (either WM, Theta, L1 or L2) to represent a layer. Generally, the first subscript (or superscript) will be used to represent the post-synaptic Unit (or the post-synaptic layer) whereas the second subscript (or superscript) will represent the pre-synaptic Unit (or the pre-synaptic layer). For instance, the symbol ${Wp}_{ij}^{L1WM}$ in the following represents an excitatory synapse from a pre-synaptic neuron at position *j* in layer “WM” to a post-synaptic neuron at position *i* in layer “L1”. Instead, when a long-range synapse is used to connect Units belonging to the same layer, only one superscript is used (e.g., ${Wp}_{ij}^{L1}$).

Briefly, “L1” receives information on the external input from “WM” through fixed ${Wp}^{L1WM}$, implements an auto-associative network through the trained synapses ${Wp}^{L1}$ and ${Wf}^{L1}$, has desynchronizing trained synapses ${Af}^{L1}$ and receives excitatory feedback from “L2” through trained ${Wp}^{L1L2}$ (this term implements the hetero-associative network). “L2” receives an input from “L1” through fixed ${Wp}^{L2L1}$. “L1” is also phase-amplitude modulated by the “Theta generator” with fixed connections ${Wp}^{L1Theta}$ (named ‘*gaintheta*’ in the following).

For each layer, E(t) and I(t) (see Eqs. *(A3)* and *(A6)*) are therefore calculated as follows:

L1:

$$\begin{aligned} {E_{i}}^{L1}\left( t \right)= \sum_{i=1}^{N} {Wp}_{ij}^{L1WM}{y_{p,j}}^{WM}\left( t-D^{L1WM} \right)+\sum_{i=1}^{N} {Wp}_{ij}^{L1L2}{y_{p,j}}^{L2}\left( t-D^{L1L2} \right) \\ +\sum_{i=1}^{N} {Wp}_{ij}^{L1}{y_{p,j}}^{L1}\left( t-D^{L1} \right) \\ +Disinhibitor \\ {I_{i}}^{L1}\left( t \right)= \sum_{i=1}^{N} {Wf}_{ij}^{L1}{y_{p,j}}^{L1}\left( t-D^{L1} \right)+\sum_{i=1}^{N} {Af}_{ij}^{L1}{z_{p,j}}^{L1}\left( t-D^{L1} \right) (A7) \\ \# \end{aligned}$$

where:

$$Disinhibitor= \mathrm{gaintheta}\left( {y_{p,j}}^{Theta}\left( t-D^{L1Theta} \right)-T \right) (A8)$$

L2:

$$\begin{aligned} {E_{i}}^{L2}\left( t \right)=\sum_{i=1}^{N} {Wp}_{ij}^{L2L1}{y_{p,j}}^{L1}\left( t-D^{L2L1} \right) (A9) \\ \# \end{aligned}$$

where *D* represents the delay in the connectivity among the layers. Specifically: 1 ms is the intra-layer (i.e., between Units belonging to the same layer) delay; 50 ms is the delay between “WM” and “L1” layers; 30 ms the delay between “L1” and “L2” layers (and, equally between “L2” and “L1”), and between “Theta generator” and “L1” layers. The values of parameters pertaining to the equations above, as well as the values of fixed synapses, are reported in the Table A4.

Eq. *(A8)* explains the global disinhibition we implemented by the “Theta generator” layer on “L1” layer. The term *‘Disinhibitor’* receives the overall activity from “Theta generator” and compares it with a threshold (T). If the global activity in “Theta generator” is below T, the inhibitor silences all Units in “L1” by acting on the inputs of the excitatory interneurons. This inhibition is then retracted as soon as the global activity in “Theta generator” rises to the threshold.

*Table A4: Long-range fixed synapses among different layer (arrays diagonal in type)*

| **Intra-unit connections** | |  |  |
| --- | --- | --- | --- |
| ${Wp}^{L1WM}$ | *100* | gaintheta | 300 |
| ${Wp}^{L2L1}$ | *300* | T | 5 |

***Training rules*** – We used Hebbian and anti-Hebbian rules to train excitatory, inhibitory, and desynchronizing synapses within the layer “L1”, and excitatory synapses from layer “L2” to layer “L1”. All excitatory and inhibitory connections are trained by means of Hebbian rule, while desynchronizing synapses are trained with an anti-Hebbian mechanism. For a more detailed description of the training procedures, we refer to the section “*Training of the network*” of the article.

Synapses within layer “L1”:

*Hebb rule (long term potentiation)*

$$\Delta{Wp}_{ij}^{L1}={\gamma_{Wp}}^{L1}\left( \frac{{z_{p,i}}^{L1}\left( t \right)}{2e_{0}}-\theta_{Wp}^{L1} \right)^{+}\left( \frac{{z_{p,j}}^{L1}\left( t \right)}{2e_{0}}-\theta_{Wp}^{L1} \right)^{+}\left( {Wp}_{max}^{L1}-{Wp}_{ij}^{L1}\left( t \right) \right)$$

*(A9)*

$${Wp}_{ij}^{L1}\left( t+T_{s} \right)= {Wp}_{ij}^{L1}(t)+ \Delta{Wp}_{ij}^{L1}(t)$$

$$\Delta{Wf}_{ij}^{L1}={\gamma_{Wf}}^{L1}\left( \frac{{z_{f,i}}^{L1}\left( t \right)}{2e_{0}}-\theta_{Wf}^{L1} \right)^{+}\left( \frac{{z_{p,j}}^{L1}\left( t \right)}{2e_{0}}-\theta_{Wf}^{L1} \right)^{+}\left( {Wf}_{max}^{L1}-{Wf}_{ij}^{L1}\left( t \right) \right)$$

*(A10)*

$${Wf}_{ij}^{L1}\left( t+T_{s} \right)= {Wf}_{ij}^{L1}(t)+ \Delta{Wf}_{ij}^{L1}(t)$$

*Anti-Hebb rule (long term potentiation)*

$$\Delta{Af}_{ij}^{L1}={\gamma_{Af}}^{L1}\left( \theta_{Af}^{L1}- \frac{{z_{f,j}}^{L1}\left( t \right)}{2e_{0}} \right)^{+}\left( \frac{{z_{p,i}}^{L1}\left( t \right)}{2e_{0}}-\theta_{Wp}^{L1} \right)^{+}\left( {Af}_{max}^{L1}-{Af}_{ij}^{L1}\left( t \right) \right)$$

*(A11)*

$${Af}_{ij}^{L1}\left( t+T_{s} \right)= {Af}_{ij}^{L1}(t)+ \Delta{Af}_{ij}^{L1}(t)$$

Synapses from “L2” layer to “L1” layer:

*Hebb rule (long term potentiation)*

$$\Delta{Wp}_{ij}^{L1L2}={\gamma_{Wp}}^{L1L2}\left( \frac{{z_{p,i}}^{L2}\left( t \right)}{2e_{0}}-\theta_{Wp}^{L1L2} \right)^{+}\left( \frac{{z_{p,j}}^{L1}\left( t \right)}{2e_{0}}-\theta_{Wp}^{L1L2} \right)^{+}\left( {Wp}_{max}^{L1L2}-{Wp}_{ij}^{L1L2}\left( t \right) \right)$$

*(A12)*

$${Wp}_{ij}^{L1L2}\left( t+T_{s} \right)= {Wp}_{ij}^{L1L2}(t)+ \Delta{Wp}_{ij}^{L1L2}(t)$$

here $\gamma$ represents the learning factor, $z_{p,i}(t)$ is the activity of the pyramidal neuron in Unit *i*, $z_{f,i}(t)$ is the activity of the fast inhibitory neuron in Unit *i*, 2*e*_0_ is the maximum firing rate (hence, all activities are normalized to the maximum). The function ()^+^ stands for the operator ‘positive part’. Finally, the last term in Eqs. (*A9-A12*) mean that the synapses cannot overcome a maximum saturation value, $W_{max}$ and so that the overall learning rate decreases approaching saturation.

*Normalization of the sum to a maximum*

After application of the Hebb rule, we checked that the sum of the synapses entering into a Unit cannot overcome a global maximum, named $W_{maxsum}$ (different for the excitatory, inhibitory and desynchronizing synapses). This avoids that an excessive input leads a Unit to saturation, eliminating the oscillatory activity (in case of excessive excitation) or moving the activity to zero (in case of excessive inhibition). We have (this example is related to $Wp$ but the same principle applies to $Wf$ and $Af$)

$$\begin{aligned} \begin{matrix} S_{i}^{Wp}=\sum_{j} {W_{p}}_{ij}. \\ If S_{i}^{Wp}> {W_{p}}_{maxsum} \Longrightarrow{W_{p}}_{ij}⟵ {W_{p}}_{ij}\cdot\frac{{W_{p}}_{maxsum}}{S_{i}^{Wp}} \end{matrix} \#\left( A13 \right) \end{aligned}$$

Values are given in Table A5.

*Numerical algorithm:*

The differential equations were numerically solved with the Euler method, with an integration step, *dt*, as low as 0.1 ms. We verified that a reduction in the integration step does not substantially improve the accuracy.

All parameters’ values are shown in following table.

*Table A5: Parameters describing the Training procedure*

| Parameter | Value |
| --- | --- |
| *dt* (ms) | 0.1 |
| *t_end_* (ms) (length of the procedure) | 250 |
| *m_p_^L1^/ m_p_^L2^* (Hz) | 4000 (current and previous episode) or 0 (others) |
| *m_f_^L1^/ m_f_^L2^* (Hz) | 200 (current and previous episode) or 0 (others) |
| $\theta$*_low_Wp_^L1^* | 0.12 |
| $\theta$_low_Wf_^L1^ | 0.8 |
| $\theta$_up_Wf_^L1^ | 0.6 |
| $\theta$_low_Wp_^L1L2^ | 0.95 |
| $\gamma_{Wp}$^L1^ | 0.5 |
| $\gamma_{Wf}$^L1^ | 0.02 |
| $\gamma_{Af}$^L1^ | 0.04 |
| $\gamma_{Wp}$^L1L2^ | 1 |
| ${Wp}_{max}^{L1}$ | 132 |
| ${Wf}_{max}^{L1}$ | 15 |
| ${Af}_{max}^{L1}$ | 1.5 |
| ${Wp}_{max}^{L1L2}$ | 90 |
| ${Wp}_{maxsum}^{L1}$ | 396 |
| ${Wf}_{maxsum}^{L1}$ | 45 |
| ${Af}_{maxsum}^{L1}$ | 105 |
| ${Wp}_{maxsum}^{L1L2}$ | 308 |

*Robustness analysis of Hebbian and anti-Hebbian learning rules:*

We tested the robustness of the networks by changing the parameters involved in synaptic training mechanisms, resulting in final synapse values up to plus or minus 10% from the basal values. The first result (Table A6) is that the network can restore up to five sequences in the same theta cycle with reasonable accuracy, even if synapse values are varied by up to 10% of their original value. The only critical case occurs by reducing the value of excitatory synapses within the “L1” layer (i.e., ${Wp}^{L1L1}$) by 10%, resulting in a compromised recovery of the first and last episodes of sequences (i.e., recovered on average only once in two times). Thus, excitatory synapses appear to play a priority role within the network, while the model appears to be more robust to variations in inhibitory (${Wf}^{L1L1}$) and desynchronizing (${Af}^{L1L1}$) synapses. Please note that when excitatory synapses (both ${Wp}^{L1L1}$and ${Wp}^{L1L2}$) are slightly increased, the behavior of the network improves in the presence of orthogonal patterns. However, over-excitation of the “L1” layer in the presence of non-orthogonal sequences results in the recovery of spurious episodes not belonging to the sequence (due to shared features, unreported in Table 3). For this reason, we decided not to increase the values of excitatory synapses in the model excessively. Furthermore, it is to be noticed that variations even more significant than 10% could be equally well governed by the model by changing the range of some parameters simultaneously (e.g., increasing both excitatory and inhibitory synapses in “L1”, or decreasing the synaptic learning factor while increasing its threshold).

*Table A6:* *Percentage of success in retrieving different episodes within a sequence, using different values for the parameters in the Hebb’s rule (Eqs. A10-A13) causing different final synapse changes. Results refer only to nonorthogonal sequences. Episodes recognized in more than 66 percent of trials are marked in green, while episodes recognized in more than 50 percent of trials (but less than 66 percent) are marked in yellow to give an immediate idea of network capacity.*

|  |  | **Episode1** | **Episode2** | **Episode3** | **Episode4** | **Episode5** |
| --- | --- | --- | --- | --- | --- | --- |
| **90%**  $\boldsymbol{Wp}^{\boldsymbol{L}\boldsymbol{1}\boldsymbol{L}\boldsymbol{1}}$ | Sequence1 | 65.0 | 100.0 | 95.0 | 95.0 | 50.0 |
|  | Sequence2 | 52.5 | 97.5 | 97.5 | 80.0 | 47.5 |
|  | Sequence3 | 55.0 | 90.0 | 100.0 | 95.0 | 40.0 |
|  | **Mean** | **57.5** | **95.83** | **97.5** | **90.0** | **45.83** |
|  |  |  |  |  |  |  |
| **110%**  $\boldsymbol{Wp}^{\boldsymbol{L}\boldsymbol{1}\boldsymbol{L}\boldsymbol{1}}$ | Sequence1 | 85.0 | 100.0 | 100.0 | 100.0 | 100.0 |
|  | Sequence2 | 87.5 | 100.0 | 100.0 | 100.0 | 97.5 |
|  | Sequence3 | 92.5 | 100.0 | 100.0 | 100.0 | 95.0 |
|  | **Mean** | **88.33** | **100.0** | **100.0** | **100.0** | **97.5** |
|  |  |  |  |  |  |  |
| **90%**  $\boldsymbol{Wf}^{\boldsymbol{L}\boldsymbol{1}\boldsymbol{L}\boldsymbol{1}}$ | Sequence1 | 77.5 | 100.0 | 100.0 | 100.0 | 97.5 |
|  | Sequence2 | 80.0 | 100.0 | 10.0 | 97.5 | 90.0 |
|  | Sequence3 | 77.5 | 100.0 | 100.0 | 95.0 | 85.0 |
|  | **Mean** | **78.33** | **100.0** | **100.0** | **97.5** | **90.83** |
|  |  |  |  |  |  |  |
| **110%**  $\boldsymbol{Wf}^{\boldsymbol{L}\boldsymbol{1}\boldsymbol{L}\boldsymbol{1}}$ | Sequence1 | 70.0 | 100.0 | 100.0 | 100.0 | 90.0 |
|  | Sequence2 | 67.5 | 100.0 | 100.0 | 97.5 | 87.5 |
|  | Sequence3 | 77.5 | 100.0 | 100.0 | 95.0 | 82.5 |
|  | **Mean** | **71.67** | **100.0** | **100.0** | **97.5** | **86.67** |
|  |  |  |  |  |  |  |
| **90%**  $\boldsymbol{Af}^{\boldsymbol{L}\boldsymbol{1}\boldsymbol{L}\boldsymbol{1}}$ | Sequence1 | 80.0 | 100.0 | 100.0 | 100.0 | 92.5 |
|  | Sequence2 | 75.0 | 100.0 | 100.0 | 97.5 | 90.0 |
|  | Sequence3 | 72.5 | 100.0 | 100.0 | 97.5 | 85.0 |
|  | **Mean** | **75.83** | **100.0** | **100.0** | **98.3** | **89.17** |
|  |  |  |  |  |  |  |
| **110%**  $\boldsymbol{Af}^{\boldsymbol{L}\boldsymbol{1}\boldsymbol{L}\boldsymbol{1}}$ | Sequence1 | 77.5 | 100.0 | 100.0 | 100.0 | 90.0 |
|  | Sequence2 | 75.0 | 100.0 | 100.0 | 97.5 | 90.0 |
|  | Sequence3 | 70.0 | 100.0 | 100.0 | 97.5 | 87.5 |
|  | **Mean** | **74.17** | **100.0** | **100.0** | **98.33** | **89.17** |
|  |  |  |  |  |  |  |
| **90%**  $\boldsymbol{Wp}^{\boldsymbol{L}\boldsymbol{1}\boldsymbol{L}\boldsymbol{2}}$ | Sequence1 | 75.0 | 100.0 | 97.5 | 97.5 | 87.5 |
|  | Sequence2 | 75.0 | 100.0 | 100.0 | 97.5 | 82.5 |
|  | Sequence3 | 70.0 | 100.0 | 100.0 | 97.5 | 80.0 |
|  | **Mean** | **77.33** | **100.0** | **99.17** | **97.5** | **83.33** |
|  |  |  |  |  |  |  |
| **110%**  $\boldsymbol{Wp}^{\boldsymbol{L}\boldsymbol{1}\boldsymbol{L}\boldsymbol{2}}$ | Sequence1 | 75.0 | 100.0 | 100.0 | 100.0 | 92.5 |
|  | Sequence2 | 77.5 | 97.5 | 100.0 | 100.0 | 97.5 |
|  | Sequence3 | 70.0 | 100.0 | 100.0 | 100.0 | 92.5 |
|  | **Mean** | **74.17** | **99.17** | **100.0** | **100.0** | **94.17** |
